# Antidepressant Mechanism Research of Acupuncture: Insights from a Genome-Wide Transcriptome Analysis of Frontal Cortex in Rats with Chronic Restraint Stress

**DOI:** 10.1155/2017/1676808

**Published:** 2017-09-26

**Authors:** Yu Wang, Huili Jiang, Hong Meng, Jing Li, XinJing Yang, Bingcong Zhao, Yang Sun, Tuya Bao

**Affiliations:** ^1^School of Acupuncture, Moxibustion and Tuina, Beijing University of Chinese Medicine, Beijing 100029, China; ^2^School of Science, Beijing Technology and Business University, Beijing 100048, China

## Abstract

Major depressive disorder (MDD) is a chronic disease that adversely affects mood and cognition. In this study, we randomly divided the rats into control group (C), model group (M), fluoxetine group (F), and acupuncture group (A), used open-field test to ascertain whether acupuncture affects chronic restraint stress (CRS) induced depression-like behaviors of rats, and explored the antidepressant mechanism of acupuncture at the molecular level of transcriptome in the frontal cortex of CRS rats by RNA-sequencing (RNA-seq). According to differentially expressed genes (DEG) analysis, we identified 134, 46, and 89 response genes differentially expressed in C versus M, F versus M, and A versus M, respectively. Through Gene Ontology (GO) term enrichment analysis and Kyoto Encyclopedia of Genes and Genomes (KEGG) pathway enrichment analysis, we identified the gene sets involved in extracellular space, inflammatory response, Toll-like receptor signaling pathway, chemokine signaling pathway, and TNF signaling pathway. In this study, RNA-seq technology was used to investigate the frontal cortex genome-wide transcriptomes in depression rats under CRS, which suggested that the antidepressant effect of acupuncture is effective and has a multitarget characteristic, which may be related to amino acid metabolism and inflammatory pathways, especially the Toll-like receptor signaling pathway, TNF signaling pathway, and NF-kappa B signaling pathway.

## 1. Introduction

Major depressive disorder (MDD) is a chronic disease that adversely affects mood and cognition [1]. Morbidity and mortality associated with MDD have a significant effect on individuals with the illness and their families. A variety of brain imaging, postmortem morphometric, and other studies have shown that MDD is involved in the regulation of emotional and cognitive brain regions, such as the frontal cortex and hippocampus [2–4]. In spite of the fact that there is considerable evidence shown that the defects of multiple genes are associated with genetic susceptibility to MDD [5], the molecular basis has been elusive.

Nowadays, in the treatment of depression, it is estimated that approximately 30–50% of depressive patients have no response to the approved antidepressant treatment, which is thought to reflect a lack of specificity in targeting underlying pathological mechanism of depression [6, 7]. As an important part of Chinese medicine, acupuncture is an effective complement and alternative to antidepressant therapy [8]. So far, numerous studies have been published in the field of depression and acupuncture; most of them hold a positive attitude towards the antidepressant efficacy of acupuncture [9–13]. However, what is the molecular mechanism of acupuncture antidepressant effect?

Many studies have shown that a variety of molecular and intracellular signal transduction pathways that are closely related to inflammation and immunity are involved in the pathogenesis of depression [7, 14, 15]. In previous studies, we also found that acupuncture could significantly reverse the depression-like behavior of rats induced by chronic stress and have an effect on the molecular and intracellular signal transduction pathways that are closely related to inflammation and immunity [16–20], suggesting that acupuncture may play a role in the regulation of multiple targets in depression. Then, is there also a special mechanism for acupuncture antidepressant?

Therefore, in this study, we used open-field test to ascertain whether acupuncture affects CRS induced depression-like behaviors in rats, and explore the antidepressant mechanism of acupuncture at the molecular level of transcriptome in the frontal cortex of CRS rats by RNA-seq.

## 2. Materials and Methods

### 2.1. Animals

Forty-eight male Sprague-Dawley (SD) rats (180 ± 20 g) were used in this study. All animals were obtained from Charles River Laboratories of Beijing, China (license number SCXK (Jing) 2012-0001). In terms of the living conditions for the rats, they were housed in the cages with free access to water and food under the circadian of 12-hour light/dark (light on at 8:00 a.m. to 8:00 p.m.); ambient temperature and relative humidity were maintained at 20–25°C and 50%  ±  10%. When the rats adapted to the environment 5 days later, they were randomly divided into control group (C), model group (M), fluoxetine group (F), and acupuncture group (A), 12 rats in each group. All protocols were approved by the Animal Ethics Committee, Beijing University of Chinese Medicine (permission number Kj-dw-18-20140816).

### 2.2. The Procedures of CRS

In this study, rats in C were herd-managed with 4 rats per cage, and fasting for water and food was performed from 9 a.m. to 3 p.m. every day. Each rat in M, F, and A was socially isolated by placing it in a separate cage and suffered improved CRS stimulation [18, 21, 22], which restrained each rat in a self-made cylinder-shaped wire net (20.5 cm long and 6.5 cm in diameter, fixing both ends with a butterfly clip) from 9 a.m. to 3 p.m. every day, lasting 28 days.

### 2.3. Fluoxetine/Acupuncture Intervention

Each rat in F was given fluoxetine (10 mg/kg) by gavage at one hour before the CRS procedures, once a day for 28 consecutive days. Each rat in A was acupunctured on Baihui (GV20, located at midline of the head) and Yintang (GV29, located at the midpoint between the two eyes) at one hour before the CRS procedures, once a day for 28 consecutive days [18, 23]. Two sterilized disposable stainless steel needles (0.18 mm × 13 mm, Huatuo Brand, manufactured by Suzhou Medicine Co., Ltd., Suzhou, China) were inserted obliquely into each acupoint as deep as 5 mm for 20 min, once a day for 28 consecutive days.

### 2.4. Behavior Assays for Major Depressive Disorder

Whether the CRS treated rat in 28 days fell into depression-like behaviors was tested on days 0, 7, 14, 21, and 28 of the study. The open-field test was used to measure it. Four odorless plastic apparatuses with the same specifications, composed of a square arena (80 × 80 cm^2^) with a 40 cm high walls, were divided into 25 equal squares (16 × 16 cm^2^) drawn on the floor. During a 3 min observation period, the rat was gently placed at the center of the apparatus, and the crossing times (defined as at least three paws in a square) and the rearing times (defined as the rat standing upright on its hind legs) were recorded; then total scores of crossing and rearing were calculated.

### 2.5. The Frontal Cortex Dissection and Collection

Each rat was anesthetized with pentobarbital sodium (i.p., 60 mg/kg) and rapidly euthanized. Then the frontal cortex of the rat was dissected and separated depending on “The Rat Brain Stereotaxic Coordinates” [24]. The collected tissues were frozen in liquid nitrogen and stored at −80°C until processing.

### 2.6. RNA Extraction and RNA-Seq Experiment

Total RNA was treated with RQ1 DNase (Promega) to remove DNA. The quality and quantity of the purified RNA were determined by measuring the absorbance at 260 nm/280 nm (A260/A280) using SmartSpec Plus (BioRad). RNA integrity was further verified by 1.5% agarose gel electrophoresis.

For every sample, 10 *μ*g of total RNA was used for RNA-seq library preparation. Polyadenylated mRNAs were purified and concentrated with oligo (dT)-conjugated magnetic beads (invitrogen) before being used for directional RNA-seq library preparation. Purified mRNAs were iron-fragmented at 95°C followed by end repair and 5′ adaptor ligation. Then reverse transcription was performed with RT primer harboring 3′ adaptor sequence and randomized hexamer. The cDNAs were purified and amplified and PCR products corresponding to 200–500 bps were purified, quantified, and stored at −80°C until being utilised for sequencing.

For high-throughput sequencing, the libraries were prepared by following the manufacturer's instructions and applied to Illumina NextSeq 500 system with 151 nt pair-end reads (ABLife Inc., Wuhan, China).

### 2.7. RNA-Seq Raw Data Clean

Raw reads were first discarded if containing more than 2-N bases, and then reads were processed by clipping adaptor and removing low quality bases. Too short reads (less than 16 nt) were also dropped. FASTX-Toolkit (version 0.0.13) was used to get the clean reads. Quality control checks were performed by FastQC to evaluate base quality [25], GC content, sequence length distribution, and duplication level. After that, clean reads were aligned to reference genome by TopHat2 [26]. The Rattus norvegicus genome Rnor 5.0 downloaded from Ensembl was used as the reference genome. Based on gene annotation of the genome, multiple mapped reads were discarded due to their ambiguous location. Uniquely localized reads were used to calculate reads number and RPKM (reads per kilobase of exon model per million mapped reads) value of each gene [27].

The data discussed in this publication have been deposited in NCBI's Gene Expression Omnibus (GEO, https://www.ncbi.nlm.nih.gov/geo/) under the accession number GSE86392.

### 2.8. Statistical Analyses

Initial processing raw data of RNA expression profiles were conducted by using edgeR software algorithm. The data of the behavior test were analyzed using SPSS 22.0 statistical software and expressed as mean ± SEM. For each group, data at each time point were analyzed applying two-way analysis of variance (ANOVA) followed by a post-hoc Tukey's test. The differences of each test are divided into the main effect, the factors, and their interaction. The difference was statistically significant (*P* < 0.05).

## 3. Results

### 3.1. CRS Induces the Rat to Express Depression-Like Behavior

**Figure 1:**
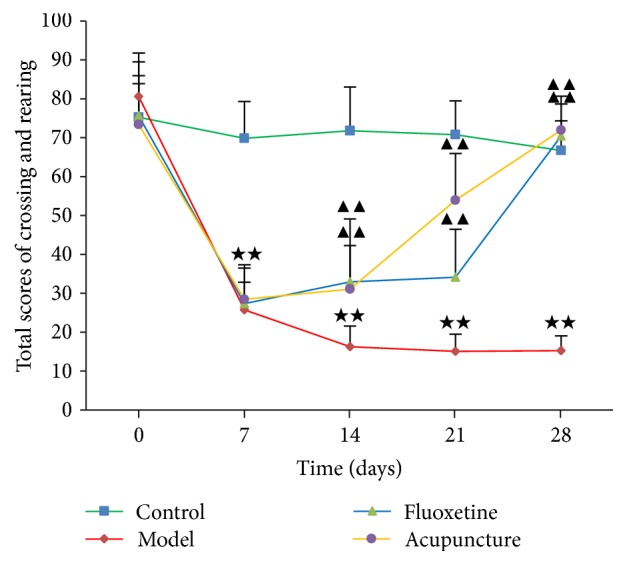
Total scores of crossing and rearing for all rats.* Notes*. ^★★^*p* < 0.01 versus C. ^▲▲^*p* < 0.01 versus M. Data are presented as mean ± SEM by two-way ANOVA.

The two-way ANOVA within days revealed total scores of crossing and rearing: the main effect [*F* = 71.446, *p* = 0.000], effect of group [*F* = 163.800, *p* = 0.000], effect of time [*F* = 127.161, *p* = 0.000], and interaction between group and time [*F* = 29.785, *p* = 0.000]. As shown in Figure 1 and Table 1, before the experiment, there was no significant group difference in total scores of crossing and rearing (*p* > 0.05). Compared to C, total scores of crossing and rearing of rats in M were significantly reduced in days 7, 14, 21, and 28 (*p* < 0.01, *p* < 0.01, *p* < 0.01, and *p* < 0.01, resp.). Compared with M, total scores of crossing and rearing in F and A were significantly increased in days 14, 21, and 28 (*p* < 0.01, *p* < 0.01, and *p* < 0.01, resp.).

### 3.2. The RNA-Seq Results

We analyzed the expression levels of frontal cortex transcriptomes, including C, M, F, and A. Based on the RPKM count data of each sample, we selected those genes that achieved a *p* value of <0.01 in differential expression analysis and submitted those genes to DAVID (Database for Annotation, Visualization, and Integrated Discovery) Functional Annotation Tool (http://david.abcc.ncifcrf.gov/summary.jsp) to annotate and enrich the genes at the levels of GO and KEGG [28].

#### 3.2.1. Differentially Expressed Genes (DEG) Analysis

**Figure 2:**
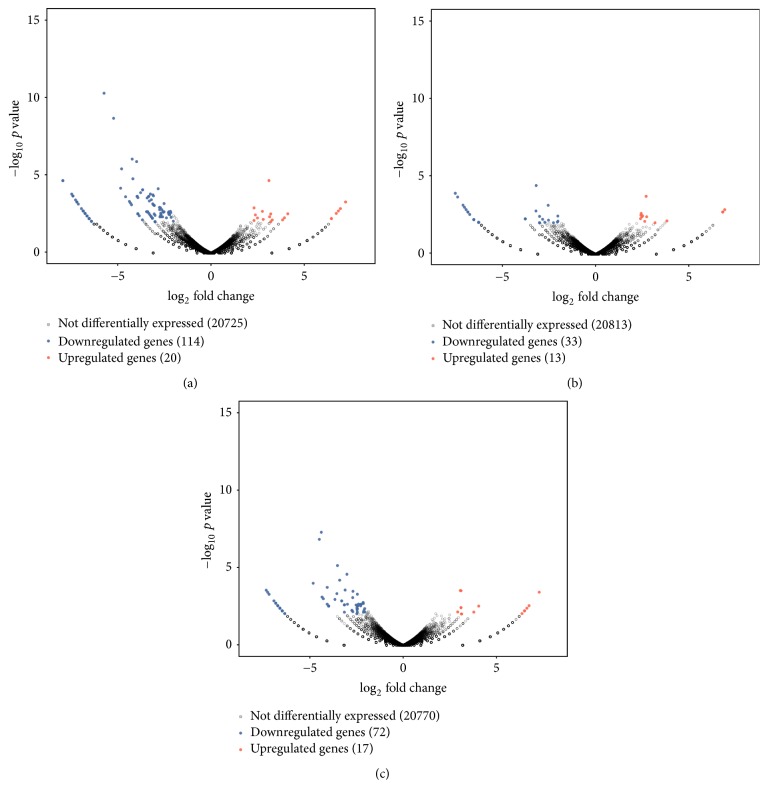
Volcano plots from pairwise comparisons of the samples. (a) Volcano plots of genes from the pairwise comparison of C versus M. (b) Volcano plots of genes from the pairwise comparison of F versus M. (c) Volcano plots of genes from the pairwise comparison of A versus M.

**Figure 3:**
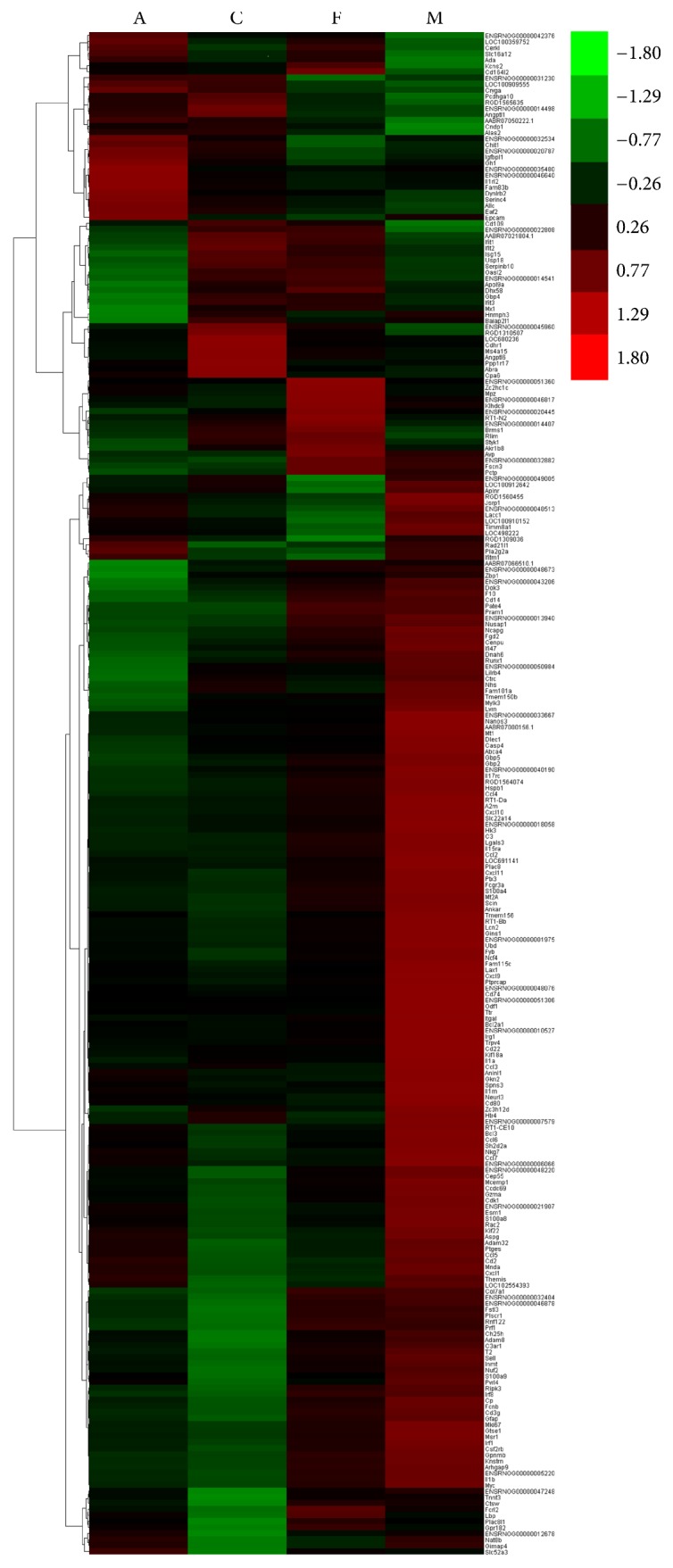
Heat map of DE genes between samples based on RPKM units. The color key represents RPKM normalized log_2_ transformed counts and each row represents a gene.

**Figure 4:**
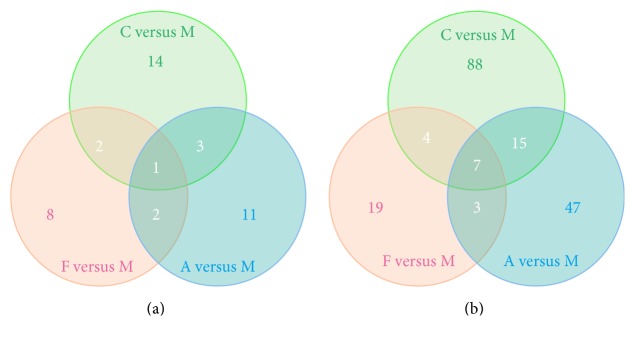
Venn diagrams showing the overlap of DE genes from pairwise comparisons of the samples. (a) Out of 41 upregulated DE genes identified as differentially expressed in at least two pairwise comparisons of the samples, 1 upregulated gene was shared across all sample pairwise comparisons. (b) Out of 183 downregulated DE genes identified as differentially expressed in at least two pairwise comparisons of the samples, 7 downregulated genes were shared across all sample pairwise comparisons.

We compared the expression of genes from C, F, and A with the expression of genes in M and analyzed the differentially expressed genes by using edgeR [29]. For each gene, significant *p* value was obtained based on the model of negative binomial distribution. Fold changes (FC) of gene expression were also estimated in the edgeR. Genes that satisfy the following two conditions are considered as differentially expressed genes: (1) the *p* value <0.01; (2) the FC level ≥2 or ≤0.05. Based on this definition, we identified 134, 46, and 89 differentially expressed (DE) genes for the respective C versus M, F versus M, and A versus M comparisons (Tables S1 and S2 in Supplementary Material available online at https://doi.org/10.1155/2017/1676808). In addition, DE genes in each pairwise comparison were divided into two parts: upregulated and downregulated genes. The pairwise comparison between C and M led to 20 upregulated and 114 downregulated DE genes, the pairwise comparison between F and M led to 13 upregulated and 33 downregulated DE genes, and the pairwise comparison between A and M led to 17 upregulated and 72 downregulated DE genes (Figure 2). In order to visualize the gene expression profiles for each group, we used Java Tree View to generate a list of genes based on thermography. As expression moves from higher to lower values, the color changes from red to green (Figure 3). In order to examine the overlap among the DE genes of each pairwise comparison, we used a Venn diagram to display the results (Figure 4). There were 8 common DE genes among C versus M, F versus M, and A versus M, including 1 upregulated gene and 7 downregulated genes; 6 common DE genes between C versus M and F versus M, including 2 upregulated and 4 downregulated genes; and 18 common DE genes between C versus M and A versus M, including 3 upregulated and 15 downregulated genes (Table 2).

#### 3.2.2. GO Term Enrichment Analysis

**Figure 5:**
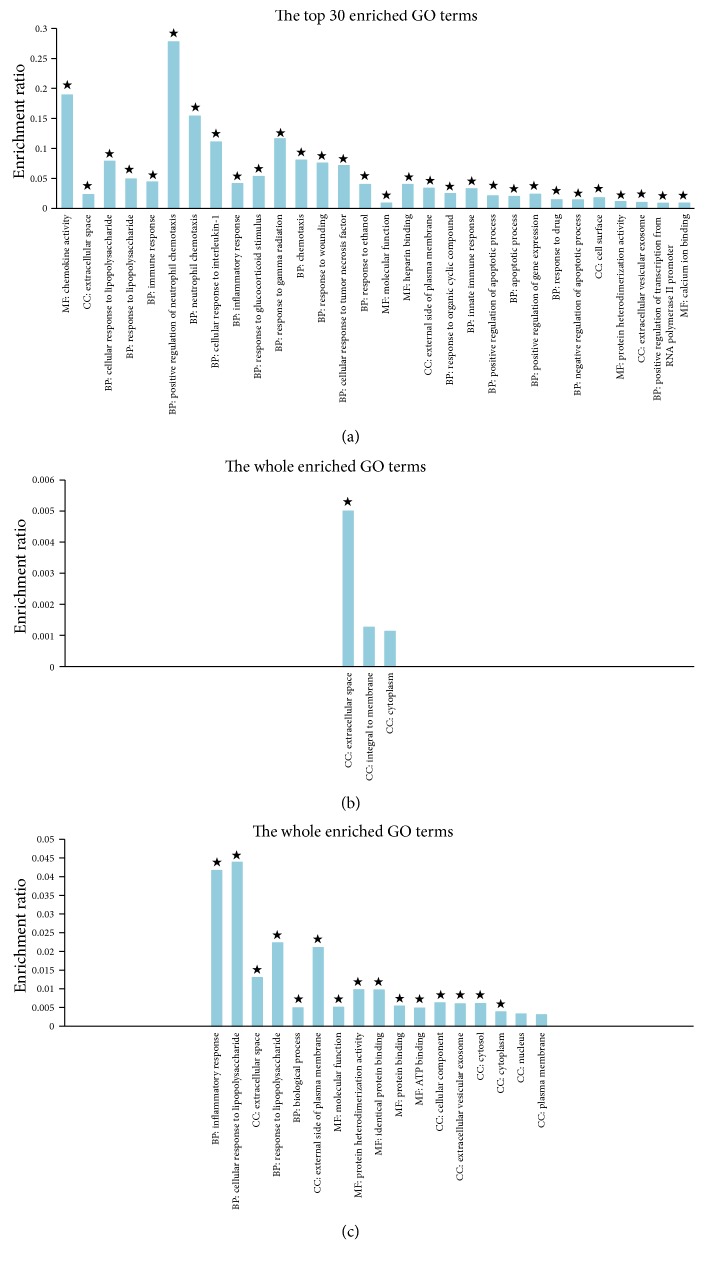
GO term enrichment analysis of DE genes from pairwise comparisons between the samples. (a) Histogram of the enriched GO terms of downregulated DE genes from the pairwise comparison of C versus M. The significance of each GO term was estimated based on corrected *p* values (corrected *p* < 0.05), which was labeled with ★ on each histogram. (b) Histogram of the enriched GO terms of downregulated DE genes from the pairwise comparison of F versus M. The significance of each GO term was estimated based on corrected *p* values (corrected *p* < 0.05), which was labeled with ★ on each histogram. (c) Histogram of the enriched GO terms of downregulated DE genes from the pairwise comparison of A versus M. The significance of each GO term was estimated based on corrected *p* values (corrected *p* value < 0.05), which was labeled with ★ on each histogram.

**Figure 6:**
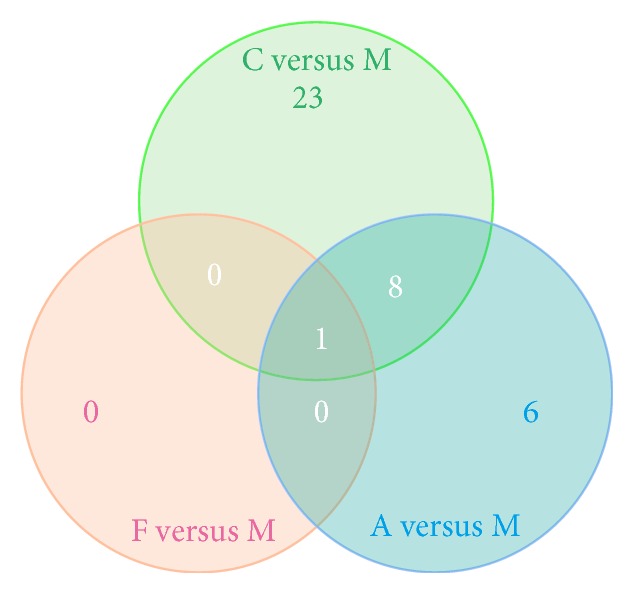
Venn diagrams showing the overlap of significant GO terms for downregulated DE genes from pairwise comparisons of the samples. Out of 38 significant GO terms of downregulated DE genes identified as differentially expressed in at least two pairwise comparisons of the samples, 1 significant GO term was shared across all sample pairwise comparisons.

GO is an international standardized gene function classification system, which used to describe the characteristics of genes and their products in any organism. Using the GO term enrichment analysis, one or a group of genes can be classified according to the three aspects of biological process (BP), molecular function (MF), and cellular component (CC). Therefore, in order to further investigate differences between the DE genes, GO term enrichment analysis was applied to search the enriched biological function items. In this analysis, GO terms with corrected *p* value < 0.05 were considered as significantly enriched. We tested the enrichment of terms for each gene list and only identified the significantly enriched GO terms in the downregulated DE genes for the C versus M, F versus M, and A versus M comparisons, followed by 32, 1, and 15 (Table S3, Figure 5). The Venn diagram to illustrate the overlap of the significantly enriched GO terms was shown in Figure 6. There was 1 GO term commonly significantly enriched in the downregulated DE genes among the three comparisons, which were of extracellular space. Notably, there were 8 GO terms commonly significantly enriched in the downregulated DE genes between the comparisons of C versus M and A versus M, mainly involved in the cellular response to lipopolysaccharide, inflammatory response, molecular function, and external side of plasma membrane (Table 3).

#### 3.2.3. KEGG Pathway Enrichment Analysis

**Figure 7:**
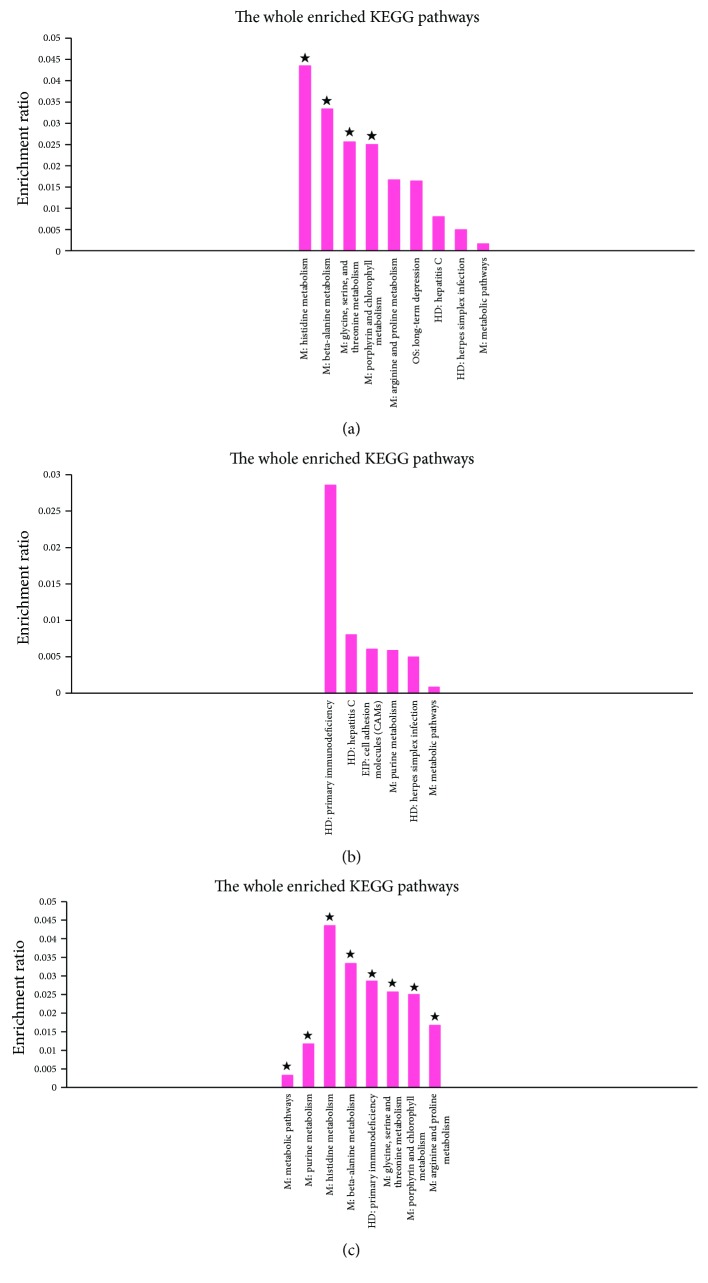
KEGG pathway enrichment analysis of upregulated DE genes from pairwise comparisons between the samples. (a) Histogram of the enriched KEGG pathways of upregulated DE genes from the pairwise comparison of C versus M. The significance of each KEGG pathway was estimated based on corrected *p* values (corrected *p* < 0.05), which was labeled with ★ on each histogram. (b) Histogram of the enriched KEGG pathways of upregulated DE genes from the pairwise comparison of F versus M. The significance of each KEGG pathway was estimated based on corrected *p* values (corrected *p* < 0.05), which was labeled with ★ on each histogram. (c) Histogram of the enriched KEGG pathways of upregulated DE genes from the pairwise comparison of A versus M. The significance of each KEGG pathway was estimated based on corrected *p* values (corrected *p* < 0.05), which was labeled with ★ on each histogram.

**Figure 8:**
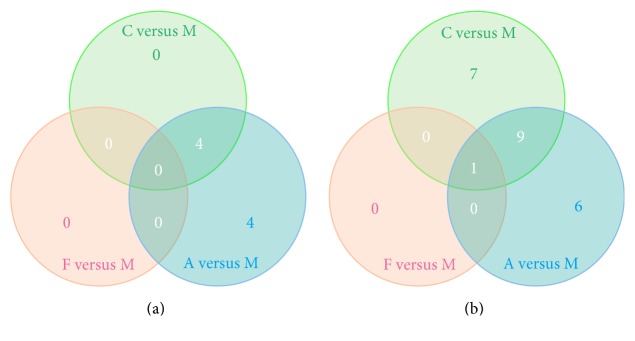
Venn diagrams showing the overlap of significant KEGG pathways of DE genes from the pairwise comparisons of the samples. (a) Out of 8 significant KEGG pathways of upregulated DE genes identified as differentially expressed in at least two pairwise comparisons of the samples, there were no significant KEGG pathways shared across all sample pairwise comparisons. (b) Out of 23 significant KEGG pathways of downregulated DE genes identified as differentially expressed in at least two pairwise comparisons of the samples, there was 1 significant KEGG pathway shared across all sample pairwise comparisons.

**Figure 9:**
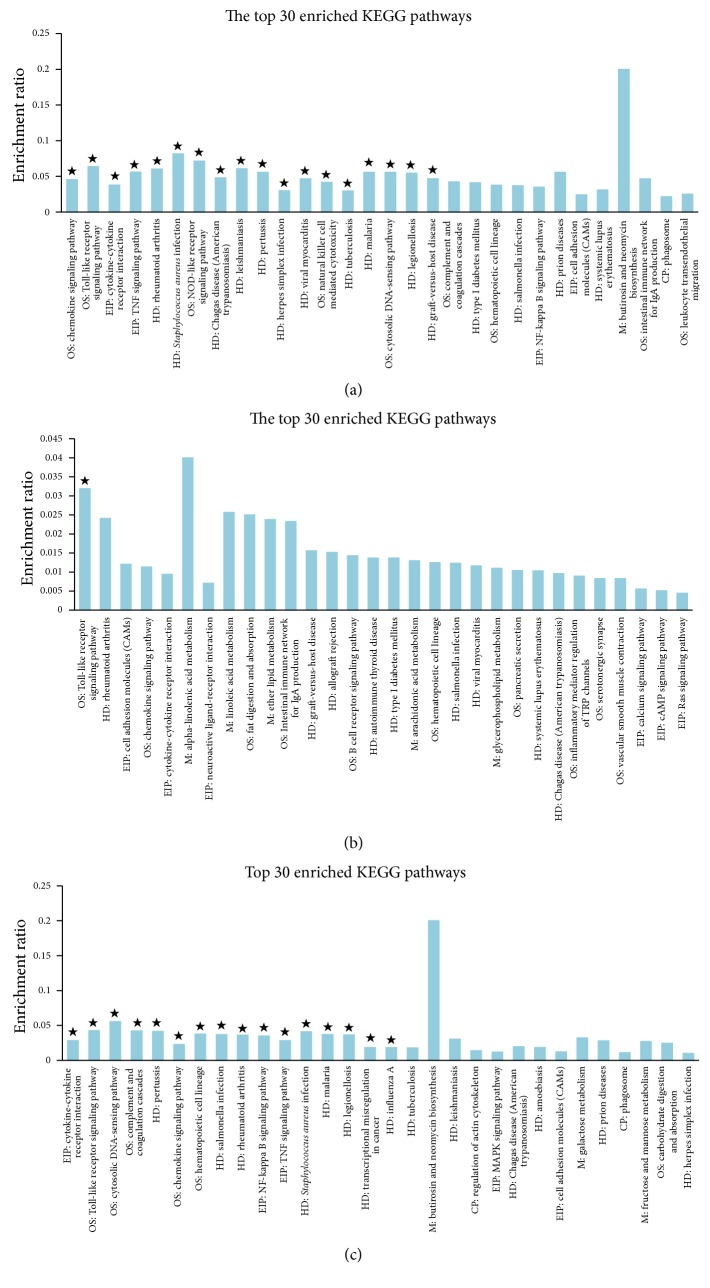
KEGG pathway enrichment analysis of downregulated DE genes from pairwise comparisons between the samples. (a) Histogram of the enriched KEGG pathways of downregulated DE genes from the pairwise comparison of C versus M. The significance of each KEGG pathway was estimated based on corrected *p* values (corrected *p* < 0.05), which was labeled with ★ on each histogram. (b) Histogram of the enriched KEGG pathways of downregulated DE genes from the pairwise comparison of F versus M. The significance of each KEGG pathway was estimated based on corrected *p* values (corrected *p* < 0.05), which was labeled with ★ on each histogram. (c) Histogram of the enriched KEGG pathways of downregulated DE genes from the pairwise comparison of A versus M. The significance of each KEGG pathway was estimated based on corrected *p* values (corrected *p* < 0.05), which was labeled with ★ on each histogram.

In an organism, the gene product does not exist in isolation, and the specific biological functions of different gene products are coordinated through the orderly coordination. KEGG is a collection of genomic and metabolic pathways, as well as database integration with new biochemical pathways. KEGG analysis is on the pathway enrichment analysis when considering the pathways topology structure and the position of each gene in the channel. The output includes pathways interest rates and the corresponding significance level (*p* value) [30, 31]. In this analysis, KEGG pathways with corrected *p* value < 0.05 were regarded as significantly enriched. For the upregulated DE genes, we finally identified 4, 0, and 8 significantly enriched KEGG pathways for the C versus M, F versus M, and A versus M comparisons, respectively (Table S4, Figure 7). The Venn diagram to illustrate the overlap of the significantly enriched KEGG pathways was shown in Figure 8(a); there were 4 KEGG pathways commonly significantly enriched in the upregulated DE genes between the comparisons of A versus M and C versus M, which were of histidine metabolism, beta-alanine metabolism, and glycine, serine, and threonine metabolism. In the downregulated DE genes, we identified 17, 1, and 16 significantly enriched KEGG pathways, respectively, for the C versus M, F versus M, and A versus M comparisons (Table S5, Figure 9). The Venn diagram to illustrate the overlap of the significantly enriched KEGG pathways was shown in Figure 8(b). There was 1 KEGG pathway commonly significantly enriched in the downregulated DE genes among the three comparisons, which was the Toll-like receptor signaling pathway. Moreover, there were 9 KEGG pathways commonly significantly enriched in the downregulated DE genes between C versus M and A versus M comparisons and which were mainly involved in chemokine signaling pathway, TNF signaling pathway, NF-kappa B signaling pathway, and cytosolic DNA-sensing pathway (Table 4).

## 4. Discussion

In this study, we used open-field test to ascertain whether acupuncture affects CRS induced depression-like behaviors in rats, and explored the antidepressant mechanism of acupuncture at the molecular level of transcriptome in the frontal cortex of CRS rats. Here, we identified some interesting findings.

Based on the results of open-field test, the ability to move and explore decreased in M, which showed that CRS successfully induced depression-like behaviors in rats. In F and A, both interventions significantly reversed these behavioral changes, indicating antidepressant effects on CRS rats.

DEG analysis showed that there were obvious differences among the three comparisons of C versus M, F versus M, and A versus M, followed by 134, 46, and 89 DE genes. In total of 269 DE genes among the three comparisons, only 8 DE genes overlapped, including Pcdhgb2, Ubd, Ttr, Cxcl9, Serpina3n, Irg1, Odf1, and Trpv4, but there are few reports of depression associated with these genes, except the Ttr [32, 33]. Notably, there were 18 common genes differentially expressed between C versus M and A versus M, and these genes are mainly involved in neurodevelopment, inflammation, signal transduction, and cellular communication; in addition, genes including Alas2 [34], C3 [35], Ccl2 [36–38], and Ptx3 [39] have been reported to be associated with depression; the others were rarely reported. These results suggest that acupuncture intervention may exert an antidepressant effect by influencing the expression of these genes in the frontal cortex.

To further clarify these differences among the three comparisons, we used GO term enrichment analysis and KEGG pathway enrichment analysis for these DE genes. In the GO enrichment analysis, there were 8 GO terms commonly significantly enriched in the downregulated DE genes between the comparisons of C versus M and A versus M, mainly involved in the cellular response to lipopolysaccharide, inflammatory response, molecular function, and external side of plasma membrane. In KEGG pathway enrichment analysis, there were 4 KEGG pathways commonly significantly enriched in the upregulated DE genes between the comparisons of A versus M and C versus M, which were of histidine metabolism, beta-alanine metabolism, and glycine, serine and threonine metabolism, and 10 KEGG pathways commonly significantly enriched in the downregulated DE genes between C versus M and A versus M comparisons and which were mainly involved in Toll-like receptor signaling pathway, chemokine signaling pathway, TNF signaling pathway, NF-kappa B signaling pathway, and cytosolic DNA-sensing pathway. These findings not only support the view that depression is associated with neuroendocrine and inflammatory pathways, but also indicate that the antidepressant effects of acupuncture may play a role in regulating amino acid metabolism and inflammatory related pathways in the frontal cortex, especially the Toll-like receptor signaling pathway, TNF signaling pathway, and NF-kappa B signaling pathway. Interestingly, the Toll-like receptor signaling pathway and NF-kappa B signaling pathway are closely related which is based on the pathway diagram provided by KEGG database; and our previous study also suggested that the antidepressant effect of acupuncture might be mediated by inhibition of inflammatory mediators via modulation of NF-kappa B in the frontal cortex [20].

## 5. Conclusion

In this study, some of the key molecular signatures coincided with important pathological features observed in previous reports; however, many molecules have not been reported in clinical and experimental studies of depression, which suggests the pathogenesis of depression is sophisticated. Further, it is precisely the pathological complexity of depression that has led to inefficiencies in the development of antidepressant drugs over the past few decades [40]. But what is astonishing is that the number of common DE genes/GO terms/KEGG pathways between the C versus M and A versus M comparisons is more than the comparisons of C versus M and F versus M, combined with the behavioral findings; it suggests the antidepressant effect of acupuncture may be achieved by targeting multiple targets. To some extent, this is in line with the view that it may be more effective to target psychosis with multiple target drugs rather than single target drugs [40]. More importantly, the multitarget antidepressant effect may be related to amino acid metabolism and inflammatory pathways, the Toll-like receptor signaling pathway and the NF-kappa B signaling pathway may be the two important pathways for acupuncture antidepressant.

However, what needs to be emphasized here is that all of the inferences above-mentioned are based on the results of this study, and the conjecture has not been verified. So, based on the scientific objectivity, we will provide more experimental evidence for antidepressant effect of acupuncture through further exploration in our next study.

## Supplementary Material

Tab.S1: Differential up regulated genes expression within the frontal cortex by edgeR.
Tab.S2: Differential down regulated genes expression within the frontal cortex by edgeR.
Tab.S3: Enriched GO terms in down regulated genes for frontal cortex.
Tab.S4: Enriched KEGG pathways in up regulated genes for frontal cortex.
Tab.S5: Enriched KEGG pathways in down regulated genes for frontal cortex.

## Figures and Tables

**Table 1:** Comparison of total scores of crossing and rearing by time ($\overset{-}{x}\pm s$, *n* = 12).

Groups	Day 0	Day 7	Day 14	Day 21	Day 28
C	75.33 ± 8.61	69.83 ± 9.50	71.83 ± 11.22	70.83 ± 8.69	66.75 ± 7.61
M	80.67 ± 11.11	25.75 ± 7.12^★★^	16.25 ± 5.36^★★^	15.08 ± 4.36^★★^	15.25 ± 3.77^★★^
F	75.67 ± 13.90	27.33 ± 9.12	32.92 ± 16.16^▲▲^	34.08 ± 12.38^▲▲^	70.50 ± 8.17^▲▲^
A	73.42 ± 12.54	28.42 ± 8.93	31.08 ± 11.21^▲▲^	53.92 ± 12.09^▲▲^	72.00 ± 8.75^▲▲^

*Notes*. ^★★^*p* < 0.01, versus C. ^▲▲^*p* < 0.01, versus M. Data are presented as mean ± SEM by two-way ANOVA.

**Table 2:** Common significant DE genes.

Samples	DE genes	
	Upregulated	Downregulated
C versus M *∩* F versus M *∩* A versus M	Pcdhgb2	Ubd, Ttr, Cxcl9, Serpina3n, Irg1, Odf1, Trpv4

F versus M *∩* C versus M	Ifit1, LOC100912860	AABR06089930.1, Cd80, Tfec, Il1rn

A versus M *∩* C versus M	Alas2, Angptl1, Cndp1	Plac8, C3, Bcl2a1, S100a4, Il15ra, Ptprcap, Lgals3, Ccl2, LOC691141, Ptx3, Itgal, Pram1, Hk3, Pate4, A2m

F versus M *∩* A versus M	Ada, Slc16a12	Hsf5, Rn50_1_2682.1, Cd22

**Table 3:** Common significant GO terms for DE genes.

Samples	GO terms	
	Upregulated	Downregulated
C versus M *∩* F versus M *∩* A versus M	—	CC: extracellular space

F versus M *∩* C versus M	—	—

A versus M *∩* C versus M	—	BP: cellular response to lipopolysaccharide BP: response to lipopolysaccharide BP: inflammatory response MF: molecular function CC: external side of plasma membrane MF: protein heterodimerization activity CC: extracellular vesicular exosome CC: cellular component

F versus M *∩* A versus M	—	—

**Table 4:** Common significant KEGG pathways for DE genes.

Samples	KEGG pathways	
	Upregulated	Downregulated
C versus M *∩* F versus M *∩* A versus M	**—**	OS: Toll-like receptor signaling pathway

F versus M *∩* C versus M	**—**	**—**

A versus M *∩* C versus M	M: histidine metabolism M: beta-alanine metabolism M: glycine, serine, and threonine metabolism M: porphyrin and chlorophyll metabolism	OS: chemokine signaling pathway EIP: cytokine-cytokine receptor interaction EIP: TNF signaling pathway HD: rheumatoid arthritis HD: *Staphylococcus aureus* infection HD: pertussis HD: malaria OS: cytosolic DNA-sensing pathway HD: legionellosis

F versus M *∩* A versus M	**—**	**—**
