# Supplementary material for: Antidepressant Mechanism Research of Acupuncture: Insights from a Genome-Wide Transcriptome Analysis of Frontal Cortex in Rats with Chronic Restraint Stress
Source: Evid Based Complement Alternat Med. 2017 Sep 26;2017:1676808. doi: 10.1155/2017/1676808 (PMC5634580; doi:10.1155/2017/1676808)
Supplement: Supplementary file 1 — Tab.S1: Differential up regulated genes expression within the frontal cortex by edgeR. Tab.S2: Differential down regulated genes expression within the frontal cortex by edgeR. Tab.S3: Enriched GO terms in down regulated genes for frontal cortex. Tab.S4: Enriched KEGG pathways in up regulated genes for frontal cortex. Tab.S5: Enriched KEGG pathways in down regulated genes for frontal cortex. [file 1676808.f1.pdf]

Tab.S1a. Differential up regulated genes expression within the frontal cortex by edgeR. log_2FC = log_2 fold change. FC = fold change.

NO.	Ensembl Gene ID	Gene Symbol	RPKM (C)	RPKM (M)	P-Value	Log_2FC
1	ENSRNOG00000019050	Ifit1	28.04	3.3	2.22E-05	3.10E+00
2	ENSRNOG00000013330	Cdhr1	5.17	1.07	1.26E-03	2.30E+00
3	ENSRNOG00000048486	LOC680236	2.89	0.18	7.45E-03	3.83E+00
4	ENSRNOG00000007999	Abra	2.66	0.39	2.13E-03	2.75E+00
5	ENSRNOG00000042990	AABR06076213.1	2.17	0.38	5.62E-03	2.49E+00
6	ENSRNOG00000000167	Alas2	2.11	0.23	4.73E-03	3.14E+00
7	ENSRNOG00000027253	Pcdhga10	2.02	0.39	3.58E-03	2.37E+00
8	ENSRNOG00000009434	RGD1310507	1.71	0.24	6.97E-03	2.79E+00
9	ENSRNOG00000022808	LOC100912860	1.39	0	2.00E-03	6.81E+00
10	ENSRNOG00000042376	Pcdhgb2	1.39	0.28	8.16E-03	2.30E+00
11	ENSRNOG00000004712	Angptl1	1.36	0	5.30E-04	7.20E+00
12	ENSRNOG00000005655	Cpa6	1.06	0.06	3.04E-03	4.11E+00
13	ENSRNOG00000027739	Cndp1	0.92	0.09	3.12E-03	3.19E+00
14	ENSRNOG00000012235	Ppp1r17	0.92	0.1	9.77E-03	3.20E+00
15	ENSRNOG00000020603	Angptl6	0.87	0	1.41E-03	6.92E+00
16	ENSRNOG00000026957	Ms4a15	0.79	0	6.15E-03	6.44E+00
17	ENSRNOG00000045960	PCDHA2	0.71	0.07	7.53E-03	3.28E+00
18	ENSRNOG00000016417	RGD1565635	0.68	0	2.86E-03	6.70E+00
19	ENSRNOG00000014498	Kbtbd12	0.49	0	6.15E-03	6.44E+00
20	ENSRNOG00000025332	Cd109	0.35	0.02	5.48E-03	3.93E+00

Tab.S1b. Differential up regulated genes expression within the frontal cortex by edgeR. log_2FC = log_2 fold change. FC = fold change.

NO.	Ensembl Gene ID	Gene Symbol	RPKM (F)	RPKM (M)	P-Value	Log_2FC
1	ENSRNOG00000019050	Ifit1	22.37	3.3	1.97E-04	2.71E+00
2	ENSRNOG00000029386	RT1-N2	4.44	0.78	2.47E-03	2.44E+00
3	ENSRNOG00000020117	LOC100911841	4.1	0.73	3.22E-03	2.43E+00
4	ENSRNOG00000009505	Cd164l2	3.4	0.32	9.77E-03	3.18E+00
5	ENSRNOG00000011369	Kcns2	2.53	0.42	3.53E-03	2.51E+00
6	ENSRNOG00000027115	Zc2hc1c	2.02	0.39	5.33E-03	2.41E+00
7	ENSRNOG00000003171	Mpz	1.9	0.27	4.05E-03	2.73E+00
8	ENSRNOG00000051360	Rn50_X_0739.1	1.77	0	1.41E-03	6.91E+00
9	ENSRNOG00000042376	Pcdhgb2	1.64	0.28	4.05E-03	2.48E+00
10	ENSRNOG00000022808	LOC100912860	1.44	0	2.00E-03	6.80E+00
11	ENSRNOG00000002824	Rlim	1.21	0.18	7.92E-03	2.63E+00
12	ENSRNOG00000010265	Ada	1.04	0.06	7.45E-03	3.82E+00
13	ENSRNOG00000021916	Slc16a12	0.68	0	2.00E-03	6.80E+00

Tab.S1c. Differential up regulated genes expression within the frontal cortex by edgeR. log_2FC = log_2 fold change. FC = fold change.

NO.	Ensembl Gene ID	Gene Symbol	RPKM (A)	RPKM (M)	P-Value	Log_2FC
1	ENSRNOG00000047746	LOC100909555	1362.66	412.28	9.40E-03	1.75E+00
2	ENSRNOG00000035480	Mir21	68	8.23	3.06E-04	3.05E+00
3	ENSRNOG00000046640	AABR06034184.1	4.13	0.34	6.15E-03	6.49E+00
4	ENSRNOG00000012450	Dynlrb2	3.76	0.56	7.29E-03	2.92E+00
5	ENSRNOG00000037091	AB041806	2.73	0.22	4.17E-03	6.62E+00
6	ENSRNOG00000042376	Pcdhgb2	2.41	0.28	3.13E-04	3.10E+00
7	ENSRNOG00000000167	Alas2	2.04	0.23	3.83E-03	3.10E+00
8	ENSRNOG00000014790	Cryga	1.5	0	6.15E-03	6.49E+00
9	ENSRNOG00000030212	AABR06024301.1	1.39	0.15	9.77E-03	3.12E+00
10	ENSRNOG00000002350	Eaf2	1.37	0.15	9.77E-03	3.12E+00
11	ENSRNOG00000010265	Ada	1.14	0.06	3.04E-03	4.04E+00
12	ENSRNOG00000008299	Allc	0.91	0.06	7.45E-03	3.78E+00
13	ENSRNOG00000004712	Angptl1	0.9	0	2.86E-03	6.73E+00
14	ENSRNOG00000021916	Slc16a12	0.85	0	3.91E-04	7.27E+00
15	ENSRNOG00000027739	Cndp1	0.83	0.09	3.83E-03	3.10E+00
16	ENSRNOG00000038241	LOC100359752	0.45	0	6.15E-03	6.49E+00
17	ENSRNOG00000015499	Serinc4	0.35	0	9.22E-03	6.36E+00

Tab.S2a. Differential down regulated genes expression within the frontal cortex by edgeR. $log_2FC = log_2$ fold change. FC = fold change.

NO.	Ensembl Gene ID	Gene Symbol	RPKM (C)	RPKM (M)	P-Value	Log_2FC
1	ENSRNOG00000002919	Gfap	121.39	439.85	6.35E-03	-1.84E+00
2	ENSRNOG00000032882	AY172581.19	29.74	118.14	9.09E-03	-2.01E+00
3	ENSRNOG00000043098	Mt2A	25.53	102.39	4.09E-03	-1.99E+00
4	ENSRNOG00000000768	Ubd	1.27	68.44	5.46E-11	-5.71E+00
5	ENSRNOG00000018735	Cd74	12.79	44.01	9.65E-03	-1.77E+00
6	ENSRNOG00000016275	Ttr	1.07	40.67	2.23E-09	-5.20E+00
7	ENSRNOG00000002217	Plac8	1.42	22.73	1.35E-06	-3.97E+00
8	ENSRNOG00000022298	Cxcl11	3.01	17.33	6.70E-04	-2.51E+00
9	ENSRNOG00000008144	Irf1	3.8	17.21	2.12E-03	-2.16E+00
10	ENSRNOG00000011913	Cp	3.92	15.19	4.93E-03	-1.94E+00
11	ENSRNOG00000046834	C3	2.1	14.99	7.47E-05	-2.82E+00
12	ENSRNOG00000022242	Cxcl9	0.64	12.19	9.30E-07	-4.21E+00
13	ENSRNOG00000047606	Bcl2a1	2.42	11.81	3.30E-03	-2.25E+00
14	ENSRNOG00000011821	S100a4	1.92	11.42	2.41E-03	-2.54E+00
15	ENSRNOG00000010906	Ccl5	1.11	10.83	2.33E-04	-3.33E+00
16	ENSRNOG00000011483	S100a9	1.19	10.54	7.85E-04	-3.08E+00
17	ENSRNOG00000008816	Gpnmb	2.2	9.91	2.60E-03	-2.15E+00
18	ENSRNOG00000017869	Irf8	1.77	8.7	2.56E-03	-2.27E+00
19	ENSRNOG00000018706	Il15ra	1.18	7.46	1.65E-03	-2.62E+00
20	ENSRNOG00000048220	Clec12a	0.33	6.39	1.71E-05	-4.17E+00
21	ENSRNOG00000021724	Ptprcap	0.56	6.19	2.91E-04	-3.41E+00
22	ENSRNOG00000011557	S100a8	0.53	6.07	2.23E-03	-3.44E+00
23	ENSRNOG00000010645	Lgals3	0.61	5.91	1.64E-04	-3.23E+00
24	ENSRNOG00000012779	Msr1	0.66	5.69	2.14E-04	-3.07E+00
25	ENSRNOG00000019141	Ch25h	0.5	5.57	8.71E-05	-3.65E+00
26	ENSRNOG00000013973	Lcn2	0.6	5.17	8.83E-04	-3.03E+00
27	ENSRNOG00000024382	Fcgr3a	0.86	4.77	2.19E-03	-2.56E+00
28	ENSRNOG00000010527	Serpina3n	0	4.65	1.25E-11	-1.04E+01
29	ENSRNOG00000030021	Ccl6	0.41	4.31	2.33E-04	-3.33E+00
30	ENSRNOG00000032708	LOC688090	0.65	4.17	1.25E-03	-2.70E+00
31	ENSRNOG00000007159	Ccl2	0.24	3.95	2.31E-04	-3.94E+00
32	ENSRNOG00000013886	Fyb	0.86	3.93	3.48E-03	-2.21E+00
33	ENSRNOG00000023473	Rnf122	0	3.76	4.17E-03	-6.62E+00
34	ENSRNOG00000003486	Mnda	0.53	3.66	1.17E-03	-2.75E+00
35	ENSRNOG00000018820	Plac8l1	0.35	3.54	4.55E-03	-3.20E+00
36	ENSRNOG00000048076	AABR06089930.1	0	3.54	6.15E-03	-6.51E+00
37	ENSRNOG00000006940	Ncf4	0.5	3.51	1.64E-03	-2.75E+00
38	ENSRNOG00000008369	Gimap4	0.59	3.17	5.55E-03	-2.38E+00
39	ENSRNOG00000033256	LOC691141	0.36	3.01	4.59E-04	-3.30E+00
40	ENSRNOG00000012280	Ptx3	0.1	2.86	3.95E-06	-4.77E+00
41	ENSRNOG00000007350	Rac2	0.49	2.82	4.64E-03	-2.66E+00

42	ENSRNOG00000006946	Arhgap9	0.41	2.43	2.25E-03	-2.51E+00
43	ENSRNOG00000002711	Nuf2	0.37	2.43	2.79E-03	-2.68E+00
44	ENSRNOG00000020465	Ripk3	0.28	2.42	8.83E-04	-3.03E+00
45	ENSRNOG00000000239	Ccl7	0	2.27	2.18E-04	-7.38E+00
46	ENSRNOG00000043416	Bcl3	0.16	2.24	1.33E-04	-3.76E+00
47	ENSRNOG00000050210	RT1-CE10	0.26	2.23	3.12E-03	-3.03E+00
48	ENSRNOG00000047248	AABR06034751.1	0.11	2.21	4.17E-03	-6.62E+00
49	ENSRNOG00000017980	Itgal	0.52	2.21	5.10E-03	-2.13E+00
50	ENSRNOG00000002802	Cxcl1	0	2.16	1.65E-04	-7.44E+00
51	ENSRNOG00000000187	Csf2rb	0.18	2.12	8.70E-05	-3.65E+00
52	ENSRNOG00000015821	Cd2	0.25	2.05	3.83E-03	-2.98E+00
53	ENSRNOG00000015851	Nat8b	0.12	2.01	3.04E-03	-3.91E+00
54	ENSRNOG00000026518	Tmem156	0.13	2	2.78E-04	-3.90E+00
55	ENSRNOG00000021907	Parvg	0.25	1.97	6.99E-04	-3.11E+00
56	ENSRNOG00000008091	Gins1	0.18	1.95	2.81E-03	-3.32E+00
57	ENSRNOG00000009342	Fcnb	0.08	1.83	4.88E-04	-4.36E+00
58	ENSRNOG00000028259	Mcemp1	0.16	1.81	2.23E-03	-3.38E+00
59	ENSRNOG00000001527	Cd80	0.18	1.79	4.55E-03	-3.20E+00
60	ENSRNOG00000032404	Top2a	0.19	1.66	1.84E-04	-3.10E+00
61	ENSRNOG00000015945	Cd3g	0	1.61	9.22E-03	-6.38E+00
62	ENSRNOG00000004500	Myc	0.24	1.6	4.05E-03	-2.69E+00
63	ENSRNOG00000011250	Inmt	0.09	1.57	3.04E-03	-3.91E+00
64	ENSRNOG00000000562	Prf1	0.24	1.54	4.75E-03	-2.59E+00
65	ENSRNOG00000039204	Pram1	0.09	1.51	6.91E-05	-4.83E+00
66	ENSRNOG00000016164	Fcrl2	0.09	1.46	2.31E-04	-3.94E+00
67	ENSRNOG00000026235	Hk3	0.16	1.46	3.82E-04	-3.19E+00
68	ENSRNOG00000017749	Nkg7	0	1.46	2.86E-03	-6.73E+00
69	ENSRNOG00000004649	Il1b	0.14	1.44	3.57E-03	-3.26E+00
70	ENSRNOG00000028137	Mki67	0.29	1.4	2.25E-03	-2.25E+00
71	ENSRNOG00000009211	C3ar1	0.06	1.34	7.97E-04	-4.24E+00
72	ENSRNOG00000046878	Shcbp1	0	1.33	2.25E-05	-7.91E+00
73	ENSRNOG00000005220	Syne2	0.14	1.29	5.84E-03	-3.13E+00
74	ENSRNOG00000009919	Irg1	0	1.28	2.25E-05	-7.91E+00
75	ENSRNOG00000006066	Tfec	0.06	1.28	6.22E-04	-4.30E+00
76	ENSRNOG00000006320	Ptges	0.07	1.26	3.04E-03	-3.91E+00
77	ENSRNOG00000039447	Pate4	0	1.25	2.00E-03	-6.83E+00
78	ENSRNOG00000012843	Aspg	0.19	1.25	3.42E-03	-2.67E+00
79	ENSRNOG00000012678	Ccnb2	0.08	1.23	7.45E-03	-3.66E+00
80	ENSRNOG00000009334	Knstrn	0	1.2	3.91E-04	-7.24E+00
81	ENSRNOG00000010603	Gzma	0	1.18	9.22E-03	-6.38E+00
82	ENSRNOG00000001975	Ugt2a3	0.14	1.17	9.77E-03	-2.99E+00
83	ENSRNOG00000020281	Kif22	0.14	1.16	9.77E-03	-2.99E+00
84	ENSRNOG00000000632	Cdk1	0	1.12	1.41E-03	-6.92E+00
85	ENSRNOG00000006578	Odf1	0.03	1.07	6.15E-03	-6.51E+00

86	ENSRNOG00000002776	Sell	0.06	1.06	3.04E-03	-3.91E+00
87	ENSRNOG00000017897	Adam8	0.04	1.02	2.44E-04	-4.56E+00
88	ENSRNOG00000022335	Rad21l1	0.11	0.99	9.77E-03	-2.99E+00
89	ENSRNOG00000010797	Esm1	0	0.92	2.18E-04	-7.38E+00
90	ENSRNOG00000027096	Ctsw	0	0.92	4.17E-03	-6.62E+00
91	ENSRNOG00000025728	Adam32	0.08	0.91	2.23E-03	-3.44E+00
92	ENSRNOG00000020332	Tnnt3	0	0.9	6.15E-03	-6.51E+00
93	ENSRNOG00000009311	Fstl3	0.1	0.84	9.77E-03	-2.99E+00
94	ENSRNOG00000005871	Il1rn	0	0.83	7.27E-04	-7.09E+00
95	ENSRNOG00000021370	Ccdc69	0	0.81	2.86E-03	-6.73E+00
96	ENSRNOG00000004498	Scin	0.04	0.7	4.07E-03	-3.83E+00
97	ENSRNOG00000020579	Col7a1	0.1	0.68	1.25E-03	-2.70E+00
98	ENSRNOG00000024172	T2	0	0.68	9.22E-03	-6.38E+00
99	ENSRNOG00000016377	Cep55	0	0.65	5.30E-04	-7.17E+00
100	ENSRNOG00000016148	Gtse1	0.07	0.65	5.84E-03	-3.13E+00
101	ENSRNOG00000014532	Lbp	0.08	0.65	9.77E-03	-2.99E+00
102	ENSRNOG00000013294	Sh2d2a	0	0.64	6.15E-03	-6.51E+00
103	ENSRNOG00000008048	Plscr1	0	0.62	6.15E-03	-6.51E+00
104	ENSRNOG00000005132	Slc52a3	0	0.59	2.00E-03	-6.83E+00
105	ENSRNOG00000004029	Pvrl4	0.06	0.59	5.84E-03	-3.13E+00
106	ENSRNOG00000028250	Lax1	0	0.57	2.00E-03	-6.83E+00
107	ENSRNOG00000014979	Themis	0	0.57	6.15E-03	-6.51E+00
108	ENSRNOG00000028896	A2m	0.08	0.56	4.77E-03	-2.73E+00
109	ENSRNOG00000015873	Spns3	0	0.55	6.15E-03	-6.51E+00
110	ENSRNOG00000004311	Gpr182	0	0.53	6.15E-03	-6.51E+00
111	ENSRNOG00000001195	Trpv4	0	0.49	5.30E-04	-7.17E+00
112	ENSRNOG00000050703	Fam115c	0	0.36	6.15E-03	-6.51E+00
113	ENSRNOG00000003991	Ankar	0	0.27	2.86E-03	-6.73E+00
114	ENSRNOG00000005780	Adamts13	0.02	0.21	9.22E-03	-6.38E+00

Tab.S2b. Differential down regulated genes expression within the frontal cortex by edgeR. log_2FC = log_2 fold change. FC = fold change.

NO.	Ensembl Gene ID	Gene Symbol	RPKM (F)	RPKM (M)	P-Value	Log_2FC
1	ENSRNOG00000000768	Ubd	17.43	68.44	3.63E-03	-2.02E+00
2	ENSRNOG00000016275	Ttr	0	40.67	3.71E-14	-1.13E+01
3	ENSRNOG00000048513	AABR06067067.1	6.15	23.29	7.76E-03	-2.01E+00
4	ENSRNOG00000033045	H2afj	3.68	14.75	8.82E-03	-2.04E+00
5	ENSRNOG00000022242	Cxcl9	2.17	12.19	7.42E-04	-2.54E+00
6	ENSRNOG00000011226	Timm8a1	0.93	5.47	9.77E-03	-3.00E+00
7	ENSRNOG00000010527	Serpina3n	0.53	4.65	4.01E-05	-3.18E+00
8	ENSRNOG00000048076	AABR06089930.1	0.25	3.54	6.15E-03	-6.52E+00
9	ENSRNOG00000019134	Htr4	0.65	3.09	9.81E-03	-2.25E+00
10	ENSRNOG00000004273	Ifitm1	0	2.15	2.86E-03	-6.74E+00

11	ENSRNOG00000009256	Gkn2	0.14	1.98	5.48E-03	-3.76E+00
12	ENSRNOG00000011205	Ccl3	0.13	1.87	5.48E-03	-3.76E+00
13	ENSRNOG00000001527	Cd80	0	1.79	2.18E-04	-7.38E+00
14	ENSRNOG00000037298	RGD1309036	0.26	1.64	9.11E-03	-2.71E+00
15	ENSRNOG00000007579	Hsf5	0.2	1.58	3.83E-03	-3.00E+00
16	ENSRNOG00000009227	Aplnr	0	1.53	1.26E-04	-7.51E+00
17	ENSRNOG00000016945	Pla2g2a	0	1.31	6.15E-03	-6.52E+00
18	ENSRNOG00000009919	Irg1	0.14	1.28	1.72E-03	-3.19E+00
19	ENSRNOG00000006066	Tfec	0.18	1.28	5.86E-03	-2.83E+00
20	ENSRNOG00000051306	Rn50_1_2682.1	0	1.25	9.22E-03	-6.26E+00
21	ENSRNOG00000030466	LOC100912642	0	1.1	1.01E-03	-7.01E+00
22	ENSRNOG00000032951	Jsrp1	0	1.08	2.00E-03	-6.83E+00
23	ENSRNOG00000006578	Odf1	0	1.07	6.15E-03	-6.52E+00
24	ENSRNOG00000015366	Neurl3	0	0.99	1.41E-03	-6.93E+00
25	ENSRNOG00000049005	LOC100125368	0.12	0.95	9.77E-03	-3.00E+00
26	ENSRNOG00000005871	Il1rn	0	0.83	7.27E-04	-7.09E+00
27	ENSRNOG00000024000	Cd22	0.13	0.75	6.66E-03	-2.52E+00
28	ENSRNOG00000022094	Lacc1	0	0.62	9.22E-03	-6.26E+00
29	ENSRNOG00000015667	Epcam	0	0.62	9.22E-03	-6.26E+00
30	ENSRNOG00000037838	RGD1560455	0	0.58	9.22E-03	-6.26E+00
31	ENSRNOG00000001195	Trpv4	0.06	0.49	9.77E-03	-3.00E+00
32	ENSRNOG00000018113	Anlnl1	0.03	0.43	2.00E-03	-6.83E+00
33	ENSRNOG00000004341	LOC498222	0	0.29	9.22E-03	-6.26E+00

Tab.S2c. Differential down regulated genes expression within the frontal cortex by edgeR. log_2FC = log_2 fold change. FC = fold change.

NO.	Ensembl Gene ID	Gene Symbol	RPKM (A)	RPKM (M)	P-Value	Log_2FC
1	ENSRNOG00000031743	Gbp2	22.23	99	1.79E-03	-2.13E+00
2	ENSRNOG00000000768	Ubd	12.46	68.44	5.24E-04	-2.44E+00
3	ENSRNOG00000016275	Ttr	1.92	40.67	5.46E-08	-4.37E+00
4	ENSRNOG00000023546	Hspb1	8.88	32.34	8.23E-03	-1.84E+00
5	ENSRNOG00000032240	Gbp5	6.99	25.63	6.70E-03	-1.86E+00
6	ENSRNOG00000002217	Plac8	1.96	22.73	7.57E-06	-3.51E+00
7	ENSRNOG00000046834	C3	1.84	14.99	2.74E-05	-3.00E+00
8	ENSRNOG00000048673	Slfn3	2.76	12.21	2.07E-03	-2.12E+00
9	ENSRNOG00000022242	Cxcl9	1.87	12.19	3.40E-04	-2.68E+00
10	ENSRNOG00000022256	Cxcl10	2.84	12.06	4.40E-03	-2.05E+00
11	ENSRNOG00000047606	Bcl2a1	3.01	11.81	9.52E-03	-1.93E+00
12	ENSRNOG00000011821	S100a4	2.58	11.42	7.51E-03	-2.12E+00
13	ENSRNOG00000032844	RT1-Da	2.89	10.79	7.96E-03	-1.91E+00
14	ENSRNOG00000002470	Ifi47	1.91	8.94	2.28E-03	-2.22E+00
15	ENSRNOG00000018706	Il15ra	0.95	7.46	2.83E-04	-3.10E+00
16	ENSRNOG00000033697	Casp4	1.45	7.07	2.91E-03	-2.27E+00

17	ENSRNOG00000033667	Lmbr1l	1.31	6.98	2.27E-03	-2.38E+00
18	ENSRNOG00000021724	Ptprcap	1.11	6.19	2.94E-03	-2.45E+00
19	ENSRNOG00000010645	Lgals3	0.55	5.91	6.58E-05	-3.39E+00
20	ENSRNOG00000010527	Serpina3n	0.2	4.65	1.56E-07	-4.48E+00
21	ENSRNOG00000033444	F10	0.7	4.25	8.68E-04	-2.69E+00
22	ENSRNOG00000006314	Zbp1	0.99	4.2	7.30E-03	-2.05E+00
23	ENSRNOG00000007159	Ccl2	0.32	3.95	4.81E-04	-3.54E+00
24	ENSRNOG00000013745	Ctrc	0.67	3.79	5.33E-03	-2.46E+00
25	ENSRNOG00000011406	Ccl4	0.4	3.7	2.55E-03	-3.14E+00
26	ENSRNOG00000013564	Dok3	0.55	3.27	2.61E-03	-2.51E+00
27	ENSRNOG00000031593	Nanos3	0.46	3.27	5.76E-03	-2.74E+00
28	ENSRNOG00000033256	LOC691141	0.52	3.01	4.03E-03	-2.47E+00
29	ENSRNOG00000012280	Ptx3	0.52	2.86	2.97E-03	-2.41E+00
30	ENSRNOG00000025764	Mt1	0.1	2.79	2.86E-03	-6.70E+00
31	ENSRNOG00000000528	Fgd2	0.64	2.77	5.82E-03	-2.09E+00
32	ENSRNOG00000017980	Itgal	0.46	2.21	2.36E-03	-2.28E+00
33	ENSRNOG00000007579	Hsf5	0.19	1.58	2.55E-03	-3.14E+00
34	ENSRNOG00000039204	Pram1	0.08	1.51	1.92E-04	-4.06E+00
35	ENSRNOG00000026235	Hk3	0	1.46	4.00E-07	-8.72E+00
36	ENSRNOG00000048193	Hnrnph3	0.06	1.41	5.30E-04	-7.16E+00
37	ENSRNOG00000002425	Pctp	0.24	1.39	9.45E-03	-2.47E+00
38	ENSRNOG00000027811	Lilrb4	0.07	1.37	3.04E-03	-3.98E+00
39	ENSRNOG00000028273	Tmem150b	0.14	1.36	1.14E-03	-3.64E+00
40	ENSRNOG00000001704	Runx1	0.13	1.29	1.42E-03	-3.28E+00
41	ENSRNOG00000009919	Irg1	0.05	1.28	1.03E-04	-4.80E+00
42	ENSRNOG00000017819	Cd14	0.06	1.28	7.97E-04	-4.33E+00
43	ENSRNOG00000039447	Pate4	0	1.25	1.41E-03	-6.90E+00
44	ENSRNOG00000051306	Rn50_1_2682.1	0	1.25	9.22E-03	-6.32E+00
45	ENSRNOG00000004921	Nusap1	0.21	1.2	7.46E-03	-2.45E+00
46	ENSRNOG00000029897	AABR06000165.1	0	1.12	2.00E-03	-6.80E+00
47	ENSRNOG00000006578	Odf1	0	1.07	4.17E-03	-6.58E+00
48	ENSRNOG00000050984	AABR06104464.1	0	1	4.17E-03	-6.58E+00
49	ENSRNOG00000027376	Il17rc	0.16	0.98	6.97E-03	-2.68E+00
50	ENSRNOG00000018058	Runx3	0	0.88	2.86E-03	-6.70E+00
51	ENSRNOG00000004575	Il1a	0.09	0.86	7.53E-03	-3.14E+00
52	ENSRNOG00000013940	RGD1565989	0	0.8	9.22E-03	-6.32E+00
53	ENSRNOG00000024000	Cd22	0.09	0.75	2.32E-03	-2.97E+00
54	ENSRNOG00000032085	Dlec1	0.03	0.63	2.29E-03	-4.06E+00
55	ENSRNOG00000022261	Cenpu	0	0.62	4.17E-03	-6.58E+00
56	ENSRNOG00000040190	AABR06077184.1	0	0.6	6.15E-03	-6.46E+00
57	ENSRNOG00000038572	Ncapg	0	0.59	2.91E-04	-7.31E+00
58	ENSRNOG00000001011	Fam101a	0	0.57	6.15E-03	-6.46E+00
59	ENSRNOG00000028896	A2m	0.05	0.56	1.42E-03	-3.28E+00
60	ENSRNOG00000049123	AABR06058376.1	0	0.55	4.17E-03	-6.58E+00

61	ENSRNOG00000042259	Slc22a14	0	0.53	4.17E-03	-6.58E+00
62	ENSRNOG00000012892	Abca4	0.09	0.52	4.57E-03	-2.44E+00
63	ENSRNOG00000001007	Baiap2l1	0	0.51	2.00E-03	-6.80E+00
64	ENSRNOG00000003950	Lvrn	0.03	0.51	3.04E-03	-3.98E+00
65	ENSRNOG00000016041	Zc3h12d	0.02	0.5	1.03E-03	-4.27E+00
66	ENSRNOG00000005037	Kif18a	0.03	0.5	3.04E-03	-3.98E+00
67	ENSRNOG00000030759	Nhs	0.07	0.5	5.76E-03	-2.74E+00
68	ENSRNOG00000001195	Trpv4	0	0.49	3.91E-04	-7.24E+00
69	ENSRNOG00000043206	AABR06018817.1	0	0.47	2.86E-03	-6.70E+00
70	ENSRNOG00000030938	RGD1564074	0	0.39	6.15E-03	-6.46E+00
71	ENSRNOG00000017546	Mylk3	0	0.34	6.15E-03	-6.46E+00
72	ENSRNOG00000015581	Dnah6	0	0.09	6.15E-03	-6.46E+00

Tab.S3a. Enriched GO terms in down regulated genes for frontal cortex (C VS. M)

NO.	Term	Input number	Background number	Enrichment Ratio	P-Value	Corrected P-Value
1	MF: chemokine activity	7	37	1.89E-01	6.33E-12	6.33E-11
2	CC: extracellular space	23	999	2.30E-02	4.14E-11	6.21E-10
3	BP: cellular response to lipopolysaccharide	9	114	7.89E-02	8.50E-11	1.11E-09
4	BP: response to lipopolysaccharide	11	224	4.91E-02	9.68E-11	1.11E-09
5	BP: immune response	10	225	4.44E-02	2.00E-09	1.11E-08
6	BP: positive regulation of neutrophil chemotaxis	5	18	2.78E-01	2.30E-09	1.11E-08
7	BP: neutrophil chemotaxis	6	39	1.54E-01	2.41E-09	1.11E-08
8	BP: cellular response to interleukin-1	6	54	1.11E-01	1.84E-08	7.05E-08
9	BP: inflammatory response	8	192	4.17E-02	1.58E-07	5.20E-07
10	BP: response to glucocorticoid stimulus	7	131	5.34E-02	1.87E-07	5.37E-07
11	BP: response to gamma radiation	5	43	1.16E-01	2.43E-07	6.22E-07
12	BP: chemotaxis	5	62	8.06E-02	1.56E-06	3.59E-06
13	BP: response to wounding	5	66	7.58E-02	2.14E-06	4.47E-06
14	BP: cellular response to tumor necrosis factor	5	70	7.14E-02	2.87E-06	5.49E-06
15	BP: response to ethanol	6	150	4.00E-02	8.07E-06	1.43E-05
16	MF: molecular_function	16	1769	9.04E-03	8.05E-06	4.03E-05
17	MF: heparin binding	5	125	4.00E-02	2.13E-05	7.10E-05
18	CC: external side of plasma membrane	8	238	3.36E-02	1.14E-05	8.57E-05
19	BP: response to organic cyclic compound	6	240	2.50E-02	1.13E-04	1.83E-04
20	BP: innate immune response	5	151	3.31E-02	1.19E-04	1.83E-04
21	BP: positive regulation of apoptotic process	6	285	2.11E-02	2.87E-04	4.13E-04
22	BP: apoptotic process	6	300	2.00E-02	3.78E-04	5.11E-04
23	BP: positive regulation of gene expression	5	210	2.38E-02	5.49E-04	7.01E-04
24	BP: response to drug	7	474	1.48E-02	7.42E-04	8.99E-04
25	BP: negative regulation of apoptotic process	6	414	1.45E-02	2.01E-03	2.31E-03
26	CC: cell surface	8	446	1.79E-02	8.69E-04	4.34E-03
27	MF: protein heterodimerization activity	6	513	1.17E-02	2.46E-03	6.15E-03
28	CC: extracellular vesicular exosome	15	1499	1.00E-02	2.51E-03	9.40E-03
29	BP: positive regulation of transcription from RNA	7	792	8.84E-03	1.27E-02	1.39E-02

	polymerase II promoter					
30	MF: calcium ion binding	6	668	8.98E-03	8.83E-03	1.77E-02
31	BP: positive regulation of transcription, DNA-dependent	5	529	9.45E-03	2.67E-02	2.79E-02
32	CC: cellular_component	14	1600	8.75E-03	1.13E-02	3.39E-02
34	MF: protein homodimerization activity	5	675	7.41E-03	3.49E-02	5.81E-02
35	CC: protein complex	6	498	1.20E-02	2.45E-02	6.11E-02
36	BP: biological_process	8	1432	5.59E-03	8.42E-02	8.42E-02
37	MF: DNA binding	6	1097	5.47E-03	7.37E-02	1.05E-01
38	CC: extracellular region	7	821	8.53E-03	7.54E-02	1.62E-01
39	MF: protein binding	6	1307	4.59E-03	1.39E-01	1.74E-01
40	MF: zinc ion binding	5	1110	4.50E-03	1.79E-01	1.99E-01
41	CC: cytosol	8	1155	6.93E-03	1.46E-01	2.74E-01
42	CC: cytoplasm	24	4417	5.43E-03	1.70E-01	2.84E-01
43	CC: nucleus	26	4919	5.29E-03	1.95E-01	2.92E-01
44	MF: ATP binding	5	1448	3.45E-03	3.55E-01	3.55E-01
45	CC: intracellular	6	1007	5.96E-03	2.98E-01	4.07E-01
46	CC: plasma membrane	16	3258	4.91E-03	3.90E-01	4.87E-01
47	CC: nucleolus	7	1724	4.06E-03	6.66E-01	7.69E-01
48	CC: integral to membrane	19	4734	4.01E-03	7.55E-01	8.09E-01

Tab.S3b. Enriched GO terms in down regulated genes for frontal cortex (F VS. M)

NO.	Term	Input number	Background number	Enrichment Ratio	P-Value	Corrected P-Value
1	CC: extracellular space	5	999	5.01E-03	7.18E-04	2.15E-03
2	CC: integral to membrane	6	4734	1.27E-03	1.41E-01	2.12E-01
3	CC: cytoplasm	5	4417	1.13E-03	2.52E-01	2.52E-01

Tab.S3c. Enriched GO terms in down regulated genes for frontal cortex (A VS. M)

NO.	Term	Input number	Background number	Enrichment Ratio	P-Value	Corrected P-Value
1	BP: inflammatory response	8	192	4.17E-02	1.02E-11	4.06E-11
2	BP: cellular response to lipopolysaccharide	5	114	4.39E-02	1.11E-07	2.21E-07
3	CC: extracellular space	13	999	1.30E-02	5.12E-07	4.09E-06
4	BP:response to lipopolysaccharide	5	224	2.23E-02	3.16E-06	4.21E-06
5	BP: biological_process	7	1432	4.89E-03	4.64E-04	4.64E-04
6	CC: external side of plasma membrane	5	238	2.10E-02	2.90E-04	1.16E-03
7	MF: molecular function	9	1769	5.09E-03	5.06E-04	1.23E-03

8	MF: protein heterodimerization activity	5	513	9.75E-03	7.17E-04	1.23E-03
9	MF: identical protein binding	5	516	9.69E-03	7.36E-04	1.23E-03
10	MF: protein binding	7	1307	5.36E-03	1.92E-03	2.40E-03
11	MF: ATP binding	7	1448	4.83E-03	3.45E-03	3.45E-03
12	CC: cellular_component	10	1600	6.25E-03	4.83E-03	1.29E-02
13	CC: extracellular vesicular exosome	9	1499	6.00E-03	9.88E-03	1.98E-02
14	CC: cytosol	7	1155	6.06E-03	2.20E-02	3.53E-02
15	CC: cytoplasm	17	4417	3.85E-03	2.93E-02	3.90E-02
16	CC: nucleus	16	4919	3.25E-03	1.30E-01	1.49E-01
17	CC: plasma membrane	10	3258	3.07E-03	2.76E-01	2.76E-01

Tab.S4a. Enriched KEGG pathways in up regulated genes for frontal cortex (C VS. M)

NO.	Term	Input number	Background number	Enrichment Ratio	P-Value	Corrected P-Value
1	M: Histidine metabolism	1	23	4.35E-02	1.27E-02	4.96E-02
2	M: beta-Alanine metabolism	1	30	3.33E-02	1.66E-02	4.96E-02
3	M: Glycine, serine and threonine metabolism	1	39	2.56E-02	2.15E-02	4.96E-02
4	M: Porphyrin and chlorophyll metabolism	1	40	2.50E-02	2.20E-02	4.96E-02
5	M: Arginine and proline metabolism	1	60	1.67E-02	3.29E-02	5.02E-02
6	OS: Long-term depression	1	61	1.64E-02	3.35E-02	5.02E-02
7	HD: Hepatitis C	1	125	8.00E-03	6.77E-02	8.70E-02
8	HD: Herpes simplex infection	1	201	4.98E-03	1.07E-01	1.21E-01
9	M: Metabolic pathways	2	1228	1.63E-03	1.37E-01	1.37E-01

Tab.S4b. Enriched KEGG pathways in up regulated genes for frontal cortex (F VS. M)

NO.	Term	Input number	Background number	Enrichment Ratio	P-Value	Corrected P-Value
1	HD: Primary immunodeficiency	1	35	2.86E-02	1.45E-02	8.71E-02
2	HD: Hepatitis C	1	125	8.00E-03	5.12E-02	9.77E-02
3	EIP: Cell adhesion molecules (CAMs)	1	166	6.02E-03	6.76E-02	9.77E-02
4	M: Purine metabolism	1	171	5.85E-03	6.96E-02	9.77E-02
5	HD: Herpes simplex infection	1	201	4.98E-03	8.15E-02	9.77E-02
6	M: Metabolic pathways	1	1228	8.14E-04	4.29E-01	4.29E-01

Tab.S4c. Enriched KEGG pathways in up regulated genes for frontal cortex (A VS. M)

NO.	Term	Input number	Background number	Enrichment Ratio	P-Value	Corrected P-Value
1	M: Metabolic pathways	4	1228	3.26E-03	8.43E-04	6.75E-03
2	M: Purine metabolism	2	171	1.17E-02	3.26E-03	1.30E-02
3	M: Histidine metabolism	1	23	4.35E-02	1.27E-02	2.52E-02
4	M: beta-Alanine metabolism	1	30	3.33E-02	1.66E-02	2.52E-02
5	HD: Primary immunodeficiency	1	35	2.86E-02	1.93E-02	2.52E-02
6	M: Glycine, serine and threonine metabolism	1	39	2.56E-02	2.15E-02	2.52E-02
7	M: Porphyrin and chlorophyll metabolism	1	40	2.50E-02	2.20E-02	2.52E-02
8	M: Arginine and proline metabolism	1	60	1.67E-02	3.29E-02	3.29E-02

Tab.S5a. Enriched KEGG pathways in down regulated genes for frontal cortex (C VS. M)

NO.	Term	Input number	Background number	Enrichment Ratio	P-Value	Corrected P-Value
1	OS: Chemokine signaling pathway	8	176	4.55E-02	1.78E-05	1.80E-03
2	OS: Toll-like receptor signaling pathway	6	94	6.38E-02	3.33E-05	1.80E-03
3	EIP: Cytokine-cytokine receptor interaction	8	212	3.77E-02	6.78E-05	1.87E-03
4	EIP: TNF signaling pathway	6	107	5.61E-02	6.92E-05	1.87E-03
5	HD: Rheumatoid arthritis	5	83	6.02E-02	2.09E-04	4.52E-03
6	HD: Staphylococcus aureus infection	4	49	8.16E-02	2.96E-04	5.32E-03
7	OS: NOD-like receptor signaling pathway	4	56	7.14E-02	4.96E-04	7.65E-03
8	HD: Chagas disease	5	104	4.81E-02	5.97E-04	8.06E-03
9	HD: Leishmaniasis	4	66	6.06E-02	9.26E-04	1.11E-02
10	HD: Pertussis	4	72	5.56E-02	1.28E-03	1.39E-02
11	HD: Herpes simplex infection	6	201	2.99E-02	2.03E-03	1.99E-02
12	HD: Viral myocarditis	4	86	4.65E-02	2.48E-03	2.23E-02
13	OS: Natural killer cell mediated cytotoxicity	4	96	4.17E-02	3.69E-03	3.06E-02
14	HD: Tuberculosis	5	169	2.96E-02	5.09E-03	3.64E-02
15	HD: Malaria	3	54	5.56E-02	5.44E-03	3.64E-02
16	OS: Cytosolic DNA-sensing pathway	3	54	5.56E-02	5.44E-03	3.64E-02
17	HD: Legionellosis	3	55	5.45E-02	5.72E-03	3.64E-02
18	HD: Graft-versus-host disease	3	64	4.69E-02	8.72E-03	5.23E-02
19	OS: Complement and coagulation cascades	3	71	4.23E-02	1.16E-02	6.58E-02
20	HD: Type I diabetes mellitus	3	73	4.11E-02	1.25E-02	6.74E-02
21	OS: Hematopoietic cell lineage	3	80	3.75E-02	1.60E-02	8.10E-02
22	HD: Salmonella infection	3	81	3.70E-02	1.65E-02	8.10E-02
23	EIP: NF-kappa B signaling pathway	3	86	3.49E-02	1.93E-02	9.08E-02
24	HD: Prion diseases	2	36	5.56E-02	2.37E-02	1.04E-01
25	EIP: Cell adhesion molecules (CAMs)	4	166	2.41E-02	2.42E-02	1.04E-01
26	HD: Systemic lupus erythematosus	3	97	3.09E-02	2.65E-02	1.10E-01
27	M: Butirosin and neomycin biosynthesis	1	5	2.00E-01	3.29E-02	1.27E-01
28	OS: Intestinal immune network for IgA production	2	43	4.65E-02	3.31E-02	1.27E-01
29	CP: Phagosome	4	185	2.16E-02	3.41E-02	1.27E-01
30	OS: Leukocyte transendothelial migration	3	119	2.52E-02	4.45E-02	1.60E-01

31	OS: Osteoclast differentiation	3	121	2.48E-02	4.63E-02	1.61E-01
32	EIP: VEGF signaling pathway	2	60	3.33E-02	6.03E-02	2.04E-01
33	HD: Colorectal cancer	2	63	3.17E-02	6.58E-02	2.13E-01
34	EIP: Jak-STAT signaling pathway	3	141	2.13E-02	6.72E-02	2.13E-01
35	HD: Allograft rejection	2	66	3.03E-02	7.13E-02	2.20E-01
36	CP: p53 signaling pathway	2	67	2.99E-02	7.32E-02	2.20E-01
37	HD: Autoimmune thyroid disease	2	73	2.74E-02	8.49E-02	2.47E-01
38	CP: Adherens junction	2	74	2.70E-02	8.69E-02	2.47E-01
39	HD: Influenza A	3	163	1.84E-02	9.42E-02	2.61E-01
40	CP: Apoptosis	2	81	2.47E-02	1.01E-01	2.68E-01
41	M: Primary bile acid biosynthesis	1	16	6.25E-02	1.02E-01	2.68E-01
42	OS: Fc gamma R-mediated phagocytosis	2	86	2.33E-02	1.12E-01	2.85E-01
43	M: Selenocompound metabolism	1	18	5.56E-02	1.14E-01	2.85E-01
44	HD: HTLV-I infection	4	285	1.40E-02	1.21E-01	2.97E-01
45	OS: Vitamin digestion and absorption	1	24	4.17E-02	1.49E-01	3.57E-01
46	EIP: Rap1 signaling pathway	3	211	1.42E-02	1.65E-01	3.82E-01
47	OS: Inflammatory mediator regulation of TRP channels	2	112	1.79E-02	1.71E-01	3.82E-01
48	CP: Regulation of actin cytoskeleton	3	216	1.39E-02	1.74E-01	3.82E-01
49	HD: Thyroid cancer	1	29	3.45E-02	1.77E-01	3.82E-01
50	HD: Viral carcinogenesis	3	218	1.38E-02	1.77E-01	3.82E-01
51	HD: Epstein-Barr virus infection	3	220	1.36E-02	1.80E-01	3.82E-01
52	M: Galactose metabolism	1	31	3.23E-02	1.88E-01	3.90E-01
53	CP: Cell cycle	2	125	1.60E-02	2.03E-01	4.13E-01
54	HD: African trypanosomiasis	1	35	2.86E-02	2.09E-01	4.16E-01
55	HD: Measles	2	130	1.54E-02	2.15E-01	4.16E-01
56	HD: Bladder cancer	1	37	2.70E-02	2.20E-01	4.16E-01
57	M: Fructose and mannose metabolism	1	37	2.70E-02	2.20E-01	4.16E-01
58	EIP: Wnt signaling pathway	2	138	1.45E-02	2.34E-01	4.26E-01
59	M: Porphyrin and chlorophyll metabolism	1	40	2.50E-02	2.35E-01	4.26E-01
60	EIP: MAPK signaling pathway	3	253	1.19E-02	2.37E-01	4.26E-01
61	OS: Carbohydrate digestion and absorption	1	41	2.44E-02	2.40E-01	4.26E-01
62	M: Tryptophan metabolism	1	45	2.22E-02	2.61E-01	4.47E-01
63	OS: Mineral absorption	1	45	2.22E-02	2.61E-01	4.47E-01
64	M: Amino sugar and nucleotide sugar metabolism	1	47	2.13E-02	2.71E-01	4.57E-01
65	HD: Type II diabetes mellitus	1	50	2.00E-02	2.85E-01	4.67E-01
66	M: Starch and sucrose metabolism	1	50	2.00E-02	2.85E-01	4.67E-01

67	HD: Transcriptional misregulation in cancer	2	162	1.23E-02	2.94E-01	4.68E-01
68	HD: Endometrial cancer	1	52	1.92E-02	2.95E-01	4.68E-01
69	HD: Acute myeloid leukemia	1	57	1.75E-02	3.18E-01	4.98E-01
70	HD: Inflammatory bowel disease (IBD)	1	59	1.69E-02	3.27E-01	5.05E-01
71	HD: Pancreatic cancer	1	65	1.54E-02	3.54E-01	5.38E-01
72	M: Glycolysis / Gluconeogenesis	1	67	1.49E-02	3.63E-01	5.44E-01
73	OS: Fc epsilon RI signaling pathway	1	69	1.45E-02	3.71E-01	5.48E-01
74	OS: B cell receptor signaling pathway	1	70	1.43E-02	3.75E-01	5.48E-01
75	HD: Chronic myeloid leukemia	1	75	1.33E-02	3.96E-01	5.63E-01
76	OS: Prolactin signaling pathway	1	75	1.33E-02	3.96E-01	5.63E-01
77	M: Arachidonic acid metabolism	1	77	1.30E-02	4.04E-01	5.67E-01
78	EIP: TGF-beta signaling pathway	1	82	1.22E-02	4.24E-01	5.82E-01
79	HD: Small cell lung cancer	1	85	1.18E-02	4.36E-01	5.82E-01
80	OS: Progesterone-mediated oocyte maturation	1	86	1.16E-02	4.39E-01	5.82E-01
81	CP: Gap junction	1	87	1.15E-02	4.43E-01	5.82E-01
82	OS: Protein digestion and absorption	1	87	1.15E-02	4.43E-01	5.82E-01
83	EIP: ErbB signaling pathway	1	88	1.14E-02	4.47E-01	5.82E-01
84	OS: Antigen processing and presentation	1	91	1.10E-02	4.58E-01	5.89E-01
85	OS: T cell receptor signaling pathway	1	105	9.52E-03	5.07E-01	6.39E-01
86	CP: Oocyte meiosis	1	108	9.26E-03	5.17E-01	6.39E-01
87	HD: Amoebiasis	1	108	9.26E-03	5.17E-01	6.39E-01
88	M: Carbon metabolism	1	110	9.09E-03	5.24E-01	6.39E-01
89	EIP: HIF-1 signaling pathway	1	111	9.01E-03	5.27E-01	6.39E-01
90	OS: Thyroid hormone signaling pathway	1	120	8.33E-03	5.55E-01	6.61E-01
91	CP: Lysosome	1	124	8.06E-03	5.67E-01	6.61E-01
92	EIP: Neuroactive ligand-receptor interaction	2	283	7.07E-03	5.68E-01	6.61E-01
93	HD: Hepatitis C	1	125	8.00E-03	5.70E-01	6.61E-01
94	OS: Axon guidance	1	127	7.87E-03	5.76E-01	6.61E-01
95	HD: Hepatitis B	1	136	7.35E-03	6.01E-01	6.79E-01
96	OS: Insulin signaling pathway	1	137	7.30E-03	6.04E-01	6.79E-01
97	CP: Signaling pathways regulating pluripotency of stem cells	1	140	7.14E-03	6.12E-01	6.80E-01
98	HD: MicroRNAs in cancer	1	142	7.04E-03	6.17E-01	6.80E-01
99	HD: Pathways in cancer	2	317	6.31E-03	6.31E-01	6.89E-01

100	EIP: Hippo signaling pathway	1	154	6.49E-03	6.47E-01	6.97E-01
101	HD: Non-alcoholic fatty liver disease (NAFLD)	1	156	6.41E-03	6.52E-01	6.97E-01
102	HD: Alzheimer's disease	1	179	5.59E-03	7.03E-01	7.44E-01
103	EIP: cAMP signaling pathway	1	195	5.13E-03	7.34E-01	7.69E-01
104	HD: Proteoglycans in cancer	1	200	5.00E-03	7.43E-01	7.71E-01
105	CP: Focal adhesion	1	205	4.88E-03	7.51E-01	7.73E-01
106	EIP: Ras signaling pathway	1	224	4.46E-03	7.82E-01	7.97E-01
107	EIP: PI3K-Akt signaling pathway	1	330	3.03E-03	8.96E-01	9.04E-01
108	M: Metabolic pathways	2	1228	1.63E-03	9.99E-01	9.99E-01

Tab.S5b. Enriched KEGG pathways in down regulated genes for frontal cortex (F VS. M)

NO.	Term	Input number	Background number	Enrichment Ratio	P-Value	Corrected P-Value
1	OS: Toll-like receptor signaling pathway	3	94	3.19E-02	1.15E-04	3.57E-03
2	HD: Rheumatoid arthritis	2	83	2.41E-02	3.52E-03	5.45E-02
3	EIP: Cell adhesion molecules (CAMs)	2	166	1.20E-02	1.35E-02	1.17E-01
4	OS: Chemokine signaling pathway	2	176	1.14E-02	1.51E-02	1.17E-01
5	EIP: Cytokine-cytokine receptor interaction	2	212	9.43E-03	2.15E-02	1.32E-01
6	M: alpha-Linolenic acid metabolism	1	25	4.00E-02	2.75E-02	1.32E-01
7	EIP: Neuroactive ligand-receptor interaction	2	283	7.07E-03	3.69E-02	1.32E-01
8	M: Linoleic acid metabolism	1	39	2.56E-02	4.25E-02	1.32E-01
9	OS: Fat digestion and absorption	1	40	2.50E-02	4.36E-02	1.32E-01
10	M: Ether lipid metabolism	1	42	2.38E-02	4.58E-02	1.32E-01
11	OS: Intestinal immune network for IgA production	1	43	2.33E-02	4.68E-02	1.32E-01
12	HD: Graft-versus-host disease	1	64	1.56E-02	6.90E-02	1.39E-01
13	HD: Allograft rejection	1	66	1.52E-02	7.11E-02	1.39E-01
14	OS: B cell receptor signaling pathway	1	70	1.43E-02	7.52E-02	1.39E-01
15	HD: Autoimmune thyroid disease	1	73	1.37E-02	7.83E-02	1.39E-01
16	HD: Type I diabetes mellitus	1	73	1.37E-02	7.83E-02	1.39E-01
17	M: Arachidonic acid metabolism	1	77	1.30E-02	8.25E-02	1.39E-01
18	OS: Hematopoietic cell lineage	1	80	1.25E-02	8.56E-02	1.39E-01
19	HD: Salmonella infection	1	81	1.23E-02	8.66E-02	1.39E-01
20	HD: Viral myocarditis	1	86	1.16E-02	9.17E-02	1.39E-01
21	M: Glycerophospholipid metabolism	1	91	1.10E-02	9.68E-02	1.39E-01

22	OS: Pancreatic secretion	1	96	1.04E-02	1.02E-01	1.39E-01
23	HD: Systemic lupus erythematosus	1	97	1.03E-02	1.03E-01	1.39E-01
24	HD: Chagas disease	1	104	9.62E-03	1.10E-01	1.42E-01
25	OS: Inflammatory mediator regulation of TRP channels	1	112	8.93E-03	1.18E-01	1.46E-01
26	OS: Serotonergic synapse	1	121	8.26E-03	1.27E-01	1.46E-01
27	OS: Vascular smooth muscle contraction	1	121	8.26E-03	1.27E-01	1.46E-01
28	EIP: Calcium signaling pathway	1	180	5.56E-03	1.83E-01	2.03E-01
29	EIP: cAMP signaling pathway	1	195	5.13E-03	1.97E-01	2.11E-01
30	EIP: Ras signaling pathway	1	224	4.46E-03	2.24E-01	2.31E-01
31	M: Metabolic pathways	1	1228	8.14E-04	7.76E-01	7.76E-01

Tab.S5c. Enriched KEGG pathways in down regulated genes for frontal cortex (A VS. M)

NO.	Term	Input number	Background number	Enrichment Ratio	P-Value	Corrected P-Value
1	EIP: Cytokine-cytokine receptor interaction	6	212	2.83E-02	2.28E-05	1.62E-03
2	OS: Toll-like receptor signaling pathway	4	94	4.26E-02	1.38E-04	4.89E-03
3	OS: Cytosolic DNA-sensing pathway	3	54	5.56E-02	4.82E-04	1.14E-02
4	OS: Complement and coagulation cascades	3	71	4.23E-02	1.08E-03	1.33E-02
5	HD: Pertussis	3	72	4.17E-02	1.12E-03	1.33E-02
6	OS: Chemokine signaling pathway	4	176	2.27E-02	1.49E-03	1.33E-02
7	OS: Hematopoietic cell lineage	3	80	3.75E-02	1.52E-03	1.33E-02
8	HD: Salmonella infection	3	81	3.70E-02	1.58E-03	1.33E-02
9	HD: Rheumatoid arthritis	3	83	3.61E-02	1.69E-03	1.33E-02
10	EIP: NF-kappa B signaling pathway	3	86	3.49E-02	1.87E-03	1.33E-02
11	EIP: TNF signaling pathway	3	107	2.80E-02	3.49E-03	2.26E-02
12	HD: Staphylococcus aureus infection	2	49	4.08E-02	8.78E-03	4.98E-02
13	HD: Malaria	2	54	3.70E-02	1.06E-02	4.98E-02
14	HD: Legionellosis	2	55	3.64E-02	1.10E-02	4.98E-02
15	HD: Transcriptional misregulation in cancer	3	162	1.85E-02	1.10E-02	4.98E-02
16	HD: Influenza A	3	163	1.84E-02	1.12E-02	4.98E-02
17	HD: Tuberculosis	3	169	1.78E-02	1.24E-02	5.17E-02
18	M: Butirosin and neomycin biosynthesis	1	5	2.00E-01	1.45E-02	5.72E-02
19	HD: Leishmaniasis	2	66	3.03E-02	1.55E-02	5.81E-02
20	CP: Regulation of actin	3	216	1.39E-02	2.38E-02	8.44E-02

	cytoskeleton					
21	EIP: MAPK signaling pathway	3	253	1.19E-02	3.57E-02	1.17E-01
22	HD: Chagas disease	2	104	1.92E-02	3.63E-02	1.17E-01
23	HD: Amoebiasis	2	108	1.85E-02	3.89E-02	1.20E-01
24	EIP: Cell adhesion molecules (CAMs)	2	166	1.20E-02	8.34E-02	2.46E-01
25	M: Galactose metabolism	1	31	3.23E-02	8.68E-02	2.46E-01
26	HD: Prion diseases	1	36	2.78E-02	1.00E-01	2.60E-01
27	CP: Phagosome	2	185	1.08E-02	1.00E-01	2.60E-01
28	M: Fructose and mannose metabolism	1	37	2.70E-02	1.03E-01	2.60E-01
29	OS: Carbohydrate digestion and absorption	1	41	2.44E-02	1.13E-01	2.69E-01
30	HD: Herpes simplex infection	2	201	9.95E-03	1.15E-01	2.69E-01
31	OS: Intestinal immune network for IgA production	1	43	2.33E-02	1.18E-01	2.69E-01
32	EIP: ABC transporters	1	46	2.17E-02	1.26E-01	2.69E-01
33	M: Amino sugar and nucleotide sugar metabolism	1	47	2.13E-02	1.29E-01	2.69E-01
34	HD: Epstein-Barr virus infection	2	220	9.09E-03	1.34E-01	2.69E-01
35	HD: Type II diabetes mellitus	1	50	2.00E-02	1.36E-01	2.69E-01
36	M: Starch and sucrose metabolism	1	50	2.00E-02	1.36E-01	2.69E-01
37	OS: NOD-like receptor signaling pathway	1	56	1.79E-02	1.51E-01	2.84E-01
38	HD: Acute myeloid leukemia	1	57	1.75E-02	1.54E-01	2.84E-01
39	HD: Inflammatory bowel disease (IBD)	1	59	1.69E-02	1.59E-01	2.84E-01
40	EIP: VEGF signaling pathway	1	60	1.67E-02	1.61E-01	2.84E-01
41	OS: RIG-I-like receptor signaling pathway	1	61	1.64E-02	1.64E-01	2.84E-01
42	HD: Graft-versus-host disease	1	64	1.56E-02	1.71E-01	2.89E-01
43	M: Glycolysis / Gluconeogenesis	1	67	1.49E-02	1.79E-01	2.95E-01
44	OS: B cell receptor signaling pathway	1	70	1.43E-02	1.86E-01	2.97E-01
45	OS: Gastric acid secretion	1	72	1.39E-02	1.91E-01	2.97E-01
46	HD: Type I diabetes mellitus	1	73	1.37E-02	1.93E-01	2.97E-01
47	HD: Chronic myeloid leukemia	1	75	1.33E-02	1.98E-01	2.97E-01
48	HD: HTLV-I infection	2	285	7.02E-03	2.01E-01	2.97E-01
49	CP: Apoptosis	1	81	1.23E-02	2.12E-01	3.07E-01
50	HD: Viral myocarditis	1	86	1.16E-02	2.23E-01	3.17E-01
51	OS: Natural killer cell mediated cytotoxicity	1	96	1.04E-02	2.46E-01	3.39E-01
52	HD: Systemic lupus erythematosus	1	97	1.03E-02	2.48E-01	3.39E-01
53	M: Carbon metabolism	1	110	9.09E-03	2.77E-01	3.63E-01

54	EIP: HIF-1 signaling pathway	1	111	9.01E-03	2.79E-01	3.63E-01
55	OS: Inflammatory mediator regulation of TRP channels	1	112	8.93E-03	2.81E-01	3.63E-01
56	OS: Leukocyte transendothelial migration	1	119	8.40E-03	2.96E-01	3.67E-01
57	OS: Osteoclast differentiation	1	121	8.26E-03	3.00E-01	3.67E-01
58	OS: Vascular smooth muscle contraction	1	121	8.26E-03	3.00E-01	3.67E-01
59	HD: Measles	1	130	7.69E-03	3.18E-01	3.79E-01
60	OS: Platelet activation	1	131	7.63E-03	3.20E-01	3.79E-01
61	OS: Insulin signaling pathway	1	137	7.30E-03	3.32E-01	3.87E-01
62	EIP: Jak-STAT signaling pathway	1	141	7.09E-03	3.40E-01	3.90E-01
63	HD: Non-alcoholic fatty liver disease (NAFLD)	1	156	6.41E-03	3.69E-01	4.12E-01
64	OS: Oxytocin signaling pathway	1	157	6.37E-03	3.71E-01	4.12E-01
65	EIP: cGMP-PKG signaling pathway	1	168	5.95E-03	3.91E-01	4.28E-01
66	EIP: Calcium signaling pathway	1	180	5.56E-03	4.13E-01	4.44E-01
67	CP: Focal adhesion	1	205	4.88E-03	4.55E-01	4.82E-01
68	EIP: Rap1 signaling pathway	1	211	4.74E-03	4.65E-01	4.86E-01
69	HD: Viral carcinogenesis	1	218	4.59E-03	4.76E-01	4.90E-01
70	HD: Pathways in cancer	1	317	3.15E-03	6.12E-01	6.21E-01
71	M: Metabolic pathways	1	1228	8.14E-04	9.80E-01	9.80E-01
